# From Turpentine to (−)‐Menthol: A New Approach

**DOI:** 10.1002/cssc.202500515

**Published:** 2025-07-09

**Authors:** Dominik Dylong, Johannes Panten, Bernhard Rußbüldt, Peter J. C. Hausoul, Regina Palkovits, Matthias Eisenacher

**Affiliations:** ^1^ Circular Transformation Lab TH Köln—University of Applied Sciences Campusplatz 1 51379 Leverkusen Germany; ^2^ Global Innovation Scent & Care Symrise AG Mühlenfeldstraße 1 37603 Holzminden Germany; ^3^ Institute of Technical and Macromolecular Chemistry RWTH Aachen University Worringerweg 2 52074 Aachen Germany; ^4^ Institute for a Sustainable Hydrogen Economy Forschungszentrum Jülich Wilhelm‐Johnen‐Straße 52428 Jülich Germany

**Keywords:** green chemistry, heterogeneous catalysis, menthol synthesis, terpenoids

## Abstract

The rising popularity of (−)‐menthol, as the main component of the natural mint aroma with applications in the pharmaceuticals, cosmetics, and food industries leads to a growing need for synthetic menthol production to satisfy the world demand. In this study, a novel green synthesis route for menthol is presented. The proposed method utilizes renewable starting materials and employs environmentally benign reaction conditions, making it an attractive alternative to conventional menthol synthesis methods. The starting material, 3‐carene, which can be obtained by distillation from crude sulfate turpentine, is transformed via heterogeneous hydrogenation and heterogeneous acidic isomerization to a mixture of unsaturated menthenes. The catalytic epoxidation of the menthenes with hydrogen peroxide and hydrogenation of the epoxides leads to a mixture of menthol isomers, which can be separated and recycled by established methods to obtain the desired (−)‐menthol.

## Graphical abstract

Renewable 3‐carene, which can be obtained from the pulp and paper industrial waste crude sulfate turpentine, is successfully transformed to the aroma compound menthol, utilizing a new, four‐step synthesis route. Heterogeneous hydrogenation, solid acid catalysis, and phase transfer catalysis are utilized under organic solvent‐free conditions, with hydrogen and hydrogen peroxide as the only stoichiometric reagents.

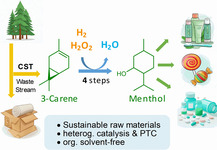

## 1 Introduction

**Scheme 1:**
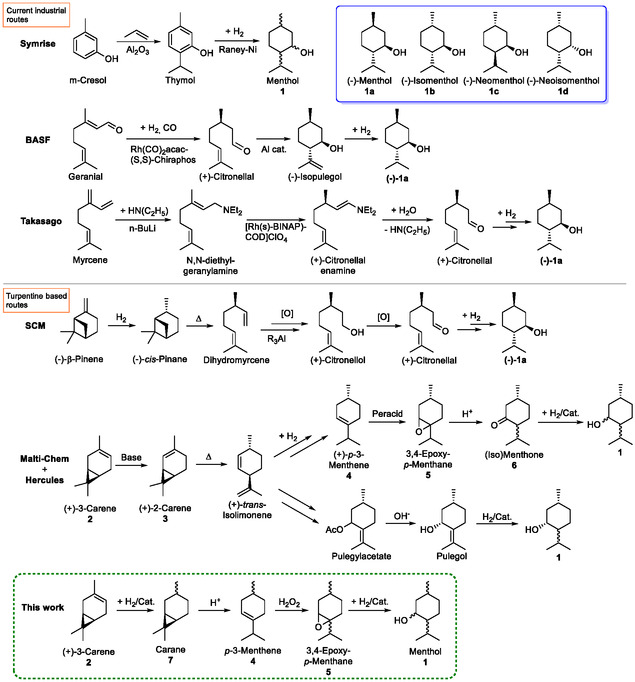
Most important industrial synthetic routes for menthol and our new synthesis route proposed in this work.

As one of the most widely used aroma compounds, (−)‐menthol is the main constituent of the fresh and cool mint aroma, already appreciated by people since ancient times. While the use of mint oil and later extracted menthol for flavoring, perfumery, and medicine has a long history,^[^ ^1^ ^]^ menthol was first characterized in the 19th century.^[^ ^2^, ^3^, ^4^, ^5^ ^]^ The applications and popularity of menthol are steadily rising.^[^ ^6^ ^]^ As the rising global demand cannot be covered solely by natural menthol, manufacturing of synthetic menthol was started in the 1970s. The main synthetic routes (**Scheme** **1**) were developed by the companies Symrise, Takasago, and BASF and use raw materials from both renewable and fossil origins. The Symrise process converts the starting materials *m*‐cresol and propene to a mixture of menthol (**1**) diastereomers in two reaction steps. The asymmetric menthol syntheses of Takasago and BASF contain isomerization and hydrogenation over chiral catalysts as the key steps, followed by the well‐known stereoselective cyclization of (+)‐citronellal. The process of Takasago starts with natural myrcene (from gum rosin or pyrolysis of *β*‐pinene), which is transformed to (−)‐menthol in five reaction steps, while the BASF process consists of three synthesis steps, starting with citral (produced from isobutene and formaldehyde). A review of these processes and the current research on menthol synthesis was recently presented by us.^[^ ^7^ ^]^ Currently, the market share of synthetic menthol is already higher than that for natural menthol. While initially the use of renewable raw materials and catalysis was motivated by lowering the production costs, the current trend of the chemical industry toward sustainability has renewed the interest for new and efficient synthesis routes for (−)‐menthol. Turpentine, of which 190 000 t/a (estimated in 2021) are generated as a byproduct in the pulp and paper industry as crude sulfate turpentine,^[^ ^8^ ^]^ is a very abundant and attractive raw material. The main components of turpentine are the bicyclic monoterpenes α‐pinene, β‐pinene, and 3‐carene (**2**).^[^ ^9^ ^]^ Because of the intrinsic high reactivity of their strained ring structures, they are interesting starting materials and can be transformed to a variety of aroma compounds.^[^ ^8^, ^10^ ^]^ In addition, they are highly available from North and East European turpentine^[^ ^8^ ^]^ and have been used for menthol production on an industrial scale in the past^[^ ^1^, ^11^ ^]^ (Scheme 1). The first process, developed by SCM Corporation,^[^ ^11^ ^]^ consists of the selective hydrogenation of (−)‐*β*‐pinene to (−)‐*cis*‐pinane, which then is pyrolyzed to dihydromyrcene. Dihydromyrcene is converted to (+)‐citronellol, either by hydroboration or hydroalumination, followed by oxidation. The catalytic oxidation of (+)‐citronellol results in (+)‐citronellal, which is finally cyclized to (−)‐menthol (**1a**). The processes of Malti‐Chem Research Center^[^ ^12^ ^]^ and Hercules Inc.^[^ ^11^, ^13^ ^]^ both start with (+)‐3‐carene (**2**), which is isomerized to 2‐carene (**3**), followed by pyrolysis to (+)‐*trans*‐isolimonene with high enantioselectivity. In the former route, isolimonene is isomerized and partially hydrogenated to (+)‐*p*‐3‐menthene (**4**), followed by epoxidation and rearrangement to menthone. In the latter route, isolimonene is transformed to pulegylacetate and saponified to pulegol. Finally, the heterogeneous hydrogenations of menthone and pulegol lead to a mixture of isomers rich in the desired (−)‐menthol (**1a**).

While the presented synthesis routes were developed for the industrial scale, they could not compete against the processes of Symrise, BASF, and Takasago and are no longer utilized. They all shared the disadvantage of containing many synthesis steps, resulting in only medium overall selectivity. Therefore, it is desirable to significantly reduce the number of synthesis steps and to implement efficient heterogeneous catalysis and green solvents and reagents. Therefore, we developed a new four‐step synthesis route (Scheme 1) for menthol utilizing (+)‐3‐carene (**2**) as a starting material.

The first step is the heterogeneous hydrogenation of **2** to carane (**7**). The highest yields for this step were reported with Ni‐^[^ ^14^, ^15^, ^16^, ^17^, ^18^ ^]^ and Pt‐^[^ ^19^, ^20^ ^]^ catalysts under high H_2_ pressures. Li‐zhi et al. achieved a yield of over 96% with 93% selectivity using Raney‐Ni at 100 °C under 90 bar H_2_ pressure.^[^ ^14^ ^]^ Hannah and McFarlin reported up to 97% carane yield with a skeletal Ni catalyst^[^ ^17^ ^]^ and up to 95% yield with Ni/SiO_2_^[^ ^18^ ^]^ in continuous operation on a pilot plant scale. The selectivity for **7** was positively influenced by high pressure and low reaction temperatures, whereas low pressures and high temperatures led to the formation of 1,1,4‐trimethylcyloheptane. The only identified stereoisomer was *cis*–**7**, which was also reported for the Pt/C catalyst.^[^ ^19^ ^]^ However, Woodroffe and Harvey determined a *cis*/*trans* ratio of 72:28 of **7** for hydrogenation over PtO_2_, with 97% chemoselectivity.^[^ ^20^ ^]^ With Pd/C, the main product was 1,1,4‐trimethylcycloheptane.^[^ ^19^, ^20^ ^]^

In the second step, **7** is isomerized to *p*‐3‐menthene (**4**). Kalechits and Rusak reported the zeolite‐catalyzed isomerization of pure *cis*‐ and *trans*‐**7** to a mixture of menthenes.^[^ ^21^ ^]^ Starting from *cis*‐**7**, the selectivity for the formation of **4** was 31% with zeolite Y at 80 °C. At this condition, cracking and disproportionation reactions were pronounced. By partial ion exchange with dysprosium cations, the selectivity was improved to 76%, while the activity of the catalyst was lowered. When *trans*‐**7** was used as a starting material, the selectivity of **4** was significantly lower (29%). For the isomerization with *p*‐TSA, a selectivity of 61% was reported for *cis*‐**7**, while *trans*‐**7** gave 31% selectivity.^[^ ^22^ ^]^ In comparison, thermal isomerization gave a maximum selectivity of 27% with *cis*‐ and 28% with *trans*‐**7** at 500 °C.

For the third step, catalytic epoxidation of **4** to 3,4‐epoxymenthane (**5**) was chosen, because of the potential use of H_2_O_2_ as a green oxidant. Thus far, reports for this reaction include only organic peracids with yields of up to 90%^[^ ^23^, ^24^, ^25^ ^]^ and methyltrioxorhenium (MTO) with 99% yield.^[^ ^26^ ^]^ An alternative approach would be a direct conversion of **4** to **1** via an asymmetric hydroboration–oxidation procedure. It was reported by Katsuhara et al. to give a mixture of (−)‐menthol (**1a**) and (−)‐isomenthol (**1b**) in a ratio of 1:1.8.^[^ ^27^ ^]^ However, hydroboration requires the stoichiometric use of expensive borane reagents and is not economical on an industrial scale.

Finally, **5** is hydrogenated to menthol. In patent literature, the hydrogenation of *cis*‐/*trans*‐**5** over Raney‐Ni at 75–78 °C under 20 bar H_2_ was reported, resulting in the preferred hydrogenation of the *cis*‐isomer to **1b**, while the *trans*‐isomer remained unreacted and was later isomerized to menthone.^[^ ^28^ ^]^

A comparison of the green chemistry metrics of the presented synthesis routes is shown in **Table** **1**. While the established industrial processes reach perfect E‐factors and atom economy, they have drawbacks. The educts of the Symrise and BASF processes are of fossil origin, with citral additionally being produced in a multistep process. On the other hand, the older turpentine‐based routes suffer from high numbers of steps and significant amounts of waste. Our proposed route combines a sustainable starting material and reagents and reduces the number of synthesis steps and generated waste. Therefore, it has the potential to become an alternative for “green” synthetic menthol production.

**Table 1:** Calculated green chemistry metrics for selected menthol synthesis routes.

	Educt	E‐factor	Atom economy	# Steps
Symrise	*m*‐Cresol + propene	0	100%	2
BASF	Citral	0	100%	3
Takasago	Myrcene	0	100%	5
*This work*	3‐Carene	0.12	90%	4
Malti‐Chem	3‐Carene	0.12	90%	7
Glidden‐Durkee	*β*‐Pinene	0.40	71%	6
Hercules	3‐Carene	1.13	44%	8

## 2 Results and Discussion

### 2.1 Hydrogenation of 3‐Carene with Heterogeneous Catalysts

The hydrogenation of (+)‐**2** was investigated using commercially available Pd, Pt, Ru, and Rh supported on activated carbon or Al_2_O_3_ with 5 wt% metal content. The catalysts were characterized by N_2_ physisorption, temperature‐programmed reduction (TPR), and CO‐pulse chemisorption, and the results are summarized in **Table** **2**.

**Table 2:** Comparison of activity and selectivity of selected hydrogenation catalysts for the hydrogenation of 3‐carene.

Catalyst	SA_BET_ [m^2^ g^−1^]		SA_NP_ [m^2^ g^−1^]	d_NP_ [nm]	H_2_ uptakea) [cm^3^ mol^−1^]
	Fresh cat.	Used cat.			
Rh/Al_2_O_3_ (A)	164	158	3.1	7.8	0b)
Rh/Al_2_O_3_ (B)	166	nd	3.9	6.2	0b)
Rh/C	810	599	12.2	2.0	10
Pt/C (A)	664	428	6.1	2.3	4.8
Pt/C (B)	776	nd	6.1	2.3	4.2
Pd/C	752	261	0.1	22	2.8
Ru/C	684	184	1.1	9.9	13

a) Hydrogen uptake normalized to active metal content.

b) Negligible hydrogen uptake. (Rh/Al_2_O_3_ (A), Merck; Rh/Al_2_O_3_ (B), Johnson Matthey; Pt/C (A), Johnson Matthey; Pt/C (B), Acros).

The BET surface areas (SA_BET_) of the as‐received catalysts are in the range of 600–800 m^2^ g^−1^ for active carbon supports and around 160 m^2^ g^−1^ for Al_2_O_3_ supports. The TPR profiles of the catalysts are available in the SI (Figure S1, Supporting Information). The highest H_2_ adsorption was measured for the Rh/C and Ru/C catalysts, followed by Pt/C, with the highest adsorption peaks between 400 and 500 °C. The Rh/Al_2_O_3_ catalysts show only negligible H_2_ adsorption, which indicates their complete reduction in the as‐received state. Therefore, 500 °C was chosen as a suitable reduction temperature for the pretreatment of the catalysts. Calculated from CO‐pulse chemisorption, metal nanoparticle surface areas (SA_NP_) in a range of 12.2–0.1 m^2^ g^−1^ and metal nanoparticle diameters (d_NP_) of 2 to 22 nm were observed. The values were used to maintain a constant ratio of substrate‐to‐metal surface in the following experiments.

#### 2.1.1 Activity and Selectivity of Hydrogenation Catalysts

**Figure 1:**
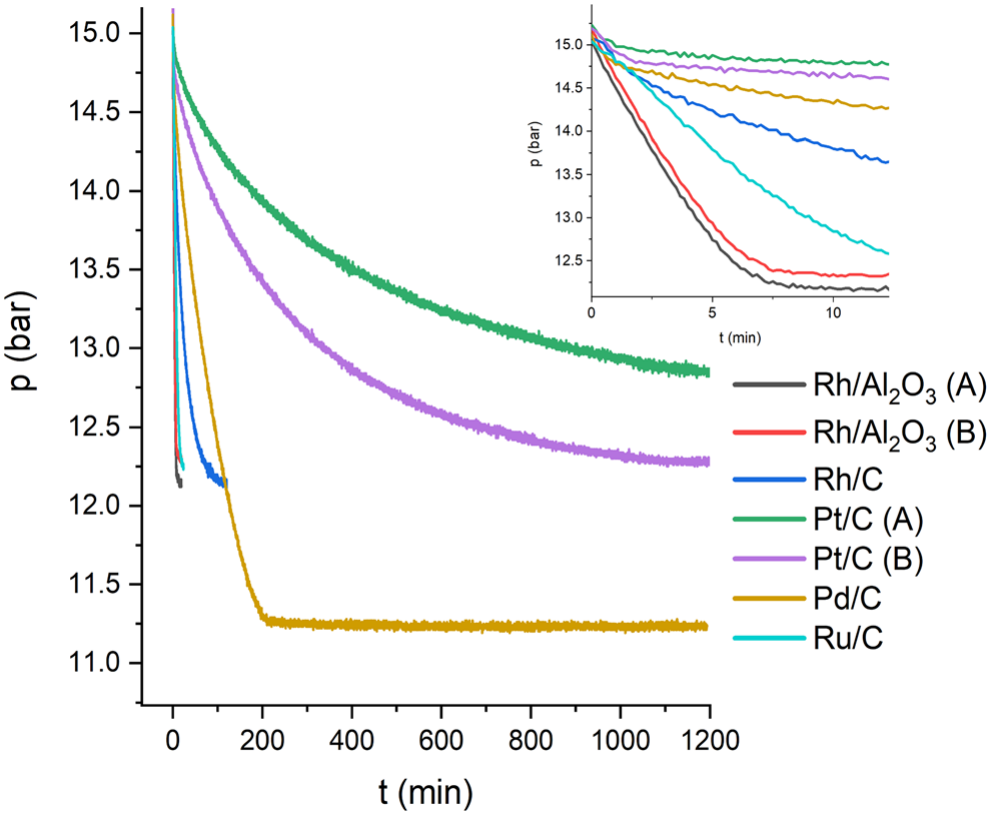
Pressure curves for the hydrogenation of 3‐carene over selected catalysts.

Preliminary experiments showed that substrate molar ratios of less than 1% and 1–10 bar H_2_ were sufficient for fast hydrogenations at room temperature. **Table** **3** shows the selectivity and reaction rates for (+)‐3‐carene (**2**) hydrogenation to carane (**7**). The substrate‐to‐metal surface area was kept constant for all experiments. The overall turnover frequency, in relation to the active metal surface atoms (TOF_ms_), gives a good measure for the reactivity of the catalysts. The Rh/Al_2_O_3_ catalysts showed the highest TOF_ms_ values of >50 min^−1^, followed by Ru/C with 36 min^−1^. With these catalysts, the reaction was pseudo‐zero order, as the reaction rate was nearly independent of the substrate concentration (see **Figure** **1** and SI, Figure S2, Supporting Information). Rh/C showed a TOF_ms_ of 8.6 min^−1^ with first‐order kinetics, while Pt/C gave low values of <1 min^−1^. With Pt, the hydrogenation appears to be second‐order in 3‐carene, resulting in incomplete conversion even after 20 h (see Figure 1). Complete hydrogenation of 3‐carene over Pt/C at 100 bar was achieved by Cocker et al. with 98% selectivity, but unfortunately, no catalyst/substrate ratio or reaction time was reported.^[^ ^19^ ^]^ Pd/C showed a medium activity with TOF_ms_ of 3 min^−1^, but the hydrogen consumption was much higher than with the other catalysts due to the pronounced formation of the cycloheptane **11,** leading to a low S_chemo_ of only 53%. Therefore, the TOF_ms_ of Pd/C cannot be directly compared with the other catalysts. The low chemoselectivity is consistent with the results reported by Cocker et al., who observed between 64% and 100% of **11** over Pd/C in propionic acid, with hydrogenolysis promoted by higher temperatures.^[^ ^19^ ^]^ In contrast, all the other catalysts in our study showed a high *S*_chemo_ of >96%, with Ru/C even reaching almost 100%.

**Table 3:** Comparison of activity and selectivity of selected hydrogenation catalysts for the hydrogenation of 3‐carene. Conditions: c(3‐carene) = 0.55 mol l^−1^; solvent: EtOH; T = 25 °C; catalyst (metal surface atoms)/substrate: 0.11 mol%; p(H_2_) = 15 bar. Abbreviations: t(max), max. reaction time; X, conversion; S, chemo‐/stereoselectivity; TON, turnover number; TOF, turnover frequency; ms, metal surface.

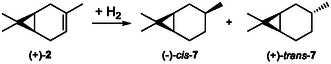				
catalyst	*X*_t(max)_ [%]	*S*_chemo_ [%]	*S*_stereo_ [%]	TOF_ms_ [min^−1^]
Rh/Al_2_O_3_ (A)	100	97	77	58
Rh/Al_2_O_3_ (B)	100	96	78	53
Rh/C	100	98	70	9
Pt/C (A)	80	96	76	0.6
Pt/C (B)	98	97	81	0.8
Pd/C	100	53	84	3
Ru/C	100	100	72	36

**Scheme 2:**
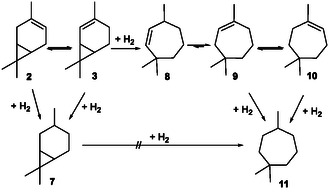
Reaction scheme of the hydrogenation of 3‐carene (**2**) to carane (**7**).

According to the reaction mechanism postulated in literature^[^ ^19^ ^]^ (**Scheme** **2**), **11** is formed as a result of the isomerization of **2** to 2‐carene (**3**), which undergoes a hydrogenolysis of the cyclopropane ring to the cycloheptenes **8**‐**10**. At high temperatures and in the absence of hydrogen, a competing mechanism involving the formation of trimethylcycloheptatrienes and –dienes could be possible through disproportionation,^[^ ^29^, ^30^ ^]^ but is unlikely under mild reaction conditions. The fast equilibration of the carenes, with ≈70% **2**% and 30% **3,** during the hydrogenation was observed by us with all catalysts, while similar values ranging from 30% to 40% of **3** were reported in literature for nickel catalysts^[^ ^31^ ^]^ and Pd/C.^[^ ^19^ ^]^ Therefore, the hydrogenolysis of **3** must be the rate‐determining step for the formation of **11**.

Small amounts of *p*‐menthanes of around 3% were identified in the reaction mixtures in all hydrogenations. While menthanes could be formed through hydrogenolysis of the external cyclopropyl bonds, the main reason was the hydrogenation of unsaturated impurities with *p*‐menthane structure present in the substrate, for example, *p*‐cymene. Further purification of the substrate reduced the amount of menthanes.

The *S*_stereo_ was highest for Pd/C with 84%, despite the low chemoselectivity. The Rh/Al_2_O_3_ and Pt/C catalysts showed similar selectivity with 77%‐81%. Rh/C and Ru/C were slightly less selective, with an *S*_stereo_ of 70% and 72%. In comparison, an *S*_stereo_ of 72% was reported for PtO_2_ in literature.^[^ ^20^ ^]^ The SA_BET_ of the spent active carbon‐supported catalysts was substantially reduced in comparison to pristine catalysts. The reduction of SA_BET_ was very high for Ru/C with 73% and Pd/C with 65%, while Pt/C and Rh/C showed a medium decrease of surface area of 36% and 26%. For the Al_2_O_3_ support, this effect was not observed, with Rh/Al_2_O_3_ showing a decrease of 3%. Overall, the highest theoretical yield of 78% *cis*‐**7** can be obtained with the catalyst Pt/C (B), but under the limitation of low activity. Rh/Al_2_O_3_ shows a slightly lower *S*_stereo_ but a very high activity even at mild conditions. This is an advantage in comparison to the reported hydrogenations over Ni catalysts, which required high pressures of above 80 bar H_2_ and temperatures above 80 °C for comparable yields.^[^ ^15^, ^17^, ^18^ ^]^ Overall, Rh and Ru catalysts showed the best performance in 3‐carene hydrogenation. Rh/Al_2_O_3_ was chosen for optimization of reaction parameters due to the highest activity and selectivity. However, due to the higher costs of this catalyst, especially ruthenium‐ and nickel‐based catalysts might be a viable alternative for this reaction.

#### 2.1.2 Influence of Reaction Parameters on the Hydrogenation of 3‐Carene with Rh/Al_2_O_3_

The influence of the temperature and H_2_ pressure on the selectivity of the hydrogenation of **2** over Rh/Al_2_O_3_ in ethanol is shown in **Table** **4**. The *S*_chemo_ declines rapidly with rising reaction temperature, similar to the observations of Cocker et al. for Pd/C.^[^ ^19^ ^]^ At 60 °C, around 40% cycloheptane **11** is formed. Surprisingly, the *S*_stereo_ for *cis*‐**7** formation slightly increases with the temperature. At 60 °C, the initially high reaction rate decreases fast after the first minutes, indicating either a deactivation of the catalyst or a change in the reaction order. Similar contrary selectivity trends were observed at varying H_2_ pressures. At 2 bar, 20% of cycloheptane **11** is formed, while at 100 bar the hydrogenolysis is mostly suppressed. At the same time, *S*_stereo_ was highest at very low pressures. As expected, the hydrogen pressure also has a significant effect on the reaction rate.

**Table 4:** Influence of temperature and H_2_ pressure on the selectivity of 3‐carene hydrogenation over Rh/Al_2_O_3_ at full conversion. Conditions: c(3‐carene) = 0.35 mol l^−1^; solvent: EtOH; catalysts: Rh/Al_2_O_3_ (5%), 0.7 mol%; reaction time: var.

T [°C]	pH_2_ [bar]	S_chemo_ [%]	S_stereo_ [%]	t [min]
25	15	96	82	14
40	15	87	85	10
60	15	57	87	70
25	2	80	88	100
25	10	96	80	10
25	100	99	79	6

No significant influence of the solvent on selectivity was found. The *S*_chemo_ in all investigated solvents is very high, with ≥99% at full conversion. The *S*_stereo_ for *cis*‐**7** formation was similar for the oxygenated solvents EtOH, MeOH, ethylacetate, acetone, and tetrahydrofuran in a range of 78%–81% and slightly lower for cyclohexane with 75% (see SI, Figure S9, Supporting Information).

The separation of the solvent from the substrate via distillation is an energy‐intensive process and not ideal from a sustainability viewpoint. Therefore, the solventless hydrogenation of 3‐carene over Rh/Al_2_O_3_ was also investigated, especially in the context of catalyst recycling. For the recycling experiments, a low catalyst‐substrate ratio of 0.1 mol% was used to emphasize the deactivation processes (for experimental details, see SI, Section 1.2.1). The catalyst showed constantly high activity in three consecutive runs, with maximum TOFs of 32–39 min^−1^ (see SI, Figure S10, Supporting Information). Overall, no significant deactivation was observed after reaching a TON of >3000 (TON_ms_: >21 500). A high chemoselectivity of >99% and a stereoselectivity for *cis*‐**7** of 76%, similar to hydrogenations in cyclohexane, were maintained for all runs.

In summary, the hydrogenation of 3‐carene over Rh/Al_2_O_3_ showed the best results at low temperature of 25 °C and medium to high H_2_ pressure above 10 bar, with carane yields of ≥96% and has successfully been conducted under solventless conditions. While similar selectivity was reported in literature for platinum and nickel catalysts, our work presents the first systematic comparison of platinum group metal catalysts for 3‐carene hydrogenation.

### 2.2 Isomerization of Carane

#### 2.2.1 Characterization of Acidic Catalysts

The isomerization of **7** to the corresponding menthene isomers was investigated over synthetic zeolites (Y, Beta, ZSM‐5, Ferrierite, Mordenite, PSH‐3) and the ion exchange resin Amberlyst 35. The surface area, pore volume, and acid site concentration of the heterogeneous catalysts were characterized via BET and NH_3_‐TPD (see **Table** **5**, SI Figure S4, Supporting Information). The structures of the zeolitic catalysts were verified via powder X‐ray diffraction (XRD) (see SI Figure S3, Supporting Information).

**Table 5:** Physicochemical properties and catalytic performance of selected heterogeneous acidic catalysts.

Catalyst	Framework type	Pore size (# T atoms)	d_pore_ [Å]	SiO_2_/Al_2_O_3_ molar ratio^[d]^	SA_BET_ [m^2^ g^−1^]	SA_micro_ [m^2^ g^−1^]	Acid density (μmol/g)	T_HT_ [°C]	X_30min_ b) [%]	S_para_ c) [%]
Y	FAU	12	7.4 × 7.4	30	715	506	589	336	100	63
ZSM‐5	MFI	10	5.1 × 5.5	23	326	187	669	374	1	56
		10	5.3 × 5.6							
Beta	BEA	12	6.6 × 6.7	360	517	376	208	329	100	54
		12	5.6 × 5.6							
PSH‐3	MWW	10	4.0 × 5.5	–	470	340	1348	376	25	56
		10	4.1 × 5.1							
Mordenite	MOR	12	6.5 × 7.0	20	398	354	1451	494	21	55
		8	2.6 × 5.7							
Ferrierite	FER	10	4.2 × 5.4	20	288	253	1483	452	1	59
		8	3.5 × 4.8							
Amberlyst 35	–	macroporous		–	38	3	5200d)	nd	44	63
MSAa)	–	–		–	–	–	–	–	6	60

a) Methanesulfonic acid (MSA).

b) Conversion after 30 min at 80 °C in c‐Hex; catalyst (acid sites)/substrate: 2.7 mol%.

c) Selectivity at 100% conversion. T = 70 °C: Y, Beta; 80 °C: ZSM‐5, PSH‐3, A35; 100 °C: Mordenite, methanesulfonic acid; 120 °C: ferrierite.

d) Value provided by the manufacturer.

Of the used heterogeneous catalysts, the zeolite Y catalysts CBV 720 and CBV 780 show the highest BET surface area of around 700 m^2^ g^−1^. In comparison, the ion exchange resin Amberlyst 35 has a surface area of only 38 m^2^ g^−1^. However, Amberlyst 35 has the highest concentration of active sites, with 5200 μmol g^−1^, followed by the zeolites Mordenite, Ferrierite, and PSH‐3 with around 1500 μmol g^−1^. The lowest acid density of 208 μmol g^−1^ was determined for zeolite Beta, which is in agreement with its low Si/Al ratio. Overall, the measured values are in a typical range for zeolite catalysts.^[^ ^32^, ^33^ ^]^ The strength of the acid sites was roughly estimated via NH_3_‐TPD. The temperature of the *high temperature* peak (T_HT_), usually assigned to strong Brønsted‐ and Lewis‐acid sites, was highest for the zeolites Mordenite and Ferrierite and lowest for zeolites Y and Beta.

#### 2.2.2 Activity and selectivity of acidic catalysts

The conversion of carane after 30 min at 80 °C and a constant substrate‐to‐active sites ratio for all catalysts is shown in Table 5 (for further data, see SI, Figure S8, Supporting Information). Full conversion was reached after 30 min with the zeolites Y and Beta. Amberlyst 35, PSH‐3, and Mordenite achieved medium conversion, while ZSM‐5 and Ferrierite showed only minimal activity under the chosen reaction conditions. No correlation between the strength of acid sites and the catalyst activity was found. The large‐pore zeolites (Y, Beta) and the macroporous ion exchange resin showed higher activity than the zeolites with medium and small pores, indicating an influence of the active sites’ accessibility on activity.

**Figure 2:**
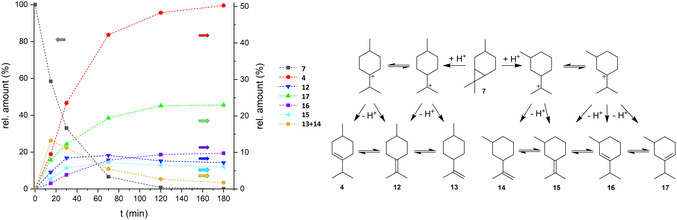
Time‐dependent distribution of products and reaction scheme of the isomerization of carane (7) over Amberlyst 35.

For the investigation of the selectivity, the relative amounts of substrate and reaction products over the course of the reaction catalyzed by Amberlyst 35 were determined via GC (**Figure** **2**). The results were in agreement with the reaction mechanism postulated by Kalechits and Rusak.^[^ ^21^ ^]^ The product mixture consisted mostly of the menthene isomers *p*‐3‐menthene (**4**), *p*‐4(8)‐menthene (**12**), *p*‐8‐menthene (**13**), *m*‐8‐menthene (**14**), *m*‐3(8)‐menthene (**15**), *m*‐2‐menthene (**16**), and *m*‐3‐menthene (**17**). In the early stages of the isomerization, the combined amount of the secondary olefins **13** and **14** was highest, with around 13%, but declined throughout the reaction to around 2%. The tertiary exocyclic olefins **12** and **15** reached their maximum amount at 70 min with 9% and 7%, respectively, and this proportion declined only a little to 7% and 6% at the end of the reaction. The amounts of the tertiary endocyclic olefins **4**, **16,** and **17** increased until the end of the reaction at 180 min and eventually reached 50%, 23%, and 10%, respectively. Additionally, *p*‐ and *m*‐menthanes and the corresponding *p*‐ and *m*‐cymene were obtained through disproportionation reactions in minor amounts of <3%. In some cases, GC results indicated product losses of around 5%, which might be attributed to the formation of oligomers. Therefore, a *S*_chemo_ for carane isomerization of >90% was generally assumed. The isomerization mechanism, according to Kalechits and Rusak, consists of two stages (Figure 2). The first stage is the scission of the cyclopropane ring under formation of tertiary carbenium ions. In this irreversible step, the stereochemistry of the formed isomers is defined. The second stage of the reaction is the proton abstraction and double bond isomerization, resulting in the formation of a mixture of menthene isomers. The double bond isomerization leads to the formation of racemates, which is unfavorable for the next steps of the synthesis route. Under mild conditions, the reaction proceeds via the formation of tertiary carbocations. Other reaction products, like cycloheptenes, *p*‐ and *m*‐1‐menthene, which could be formed through less stable secondary carbocations, were observed only under harsh reaction conditions. Filippenko et al. studied the isomerization of the *p*‐menthenes over cobalt on alumina in a temperature range of 250–350 °C.^[^ ^34^ ^]^ The reported equilibrium ratio at 250 °C was 56% **4**, 11% **12**% and 33% of *p*‐1‐menthene (see SI, Table S2, Supporting Information). In comparison, in our study, the final product mixtures showed a ratio of *p*‐isomers **4**/**12** of roughly 89:11 and a ratio of *m*‐isomers **16**/**17**/**15** of 28:57:15, with traces of **13** and **14**. Therefore, it can be assumed that the thermodynamic equilibrium was not reached under these conditions, which is beneficial for the formation of the desired *p*‐3‐menthene (**4**). The selectivity for the formation of *p*‐menthenes (*S*_para_) with Amberlyst 35 was 63% and 52% for **4**, comparable to isomerization with homogeneous methanesulfonic acid. For the microporous zeolite catalysts, an influence of the pore size on the product distribution (“shape selectivity”) was expected. However, the selectivity was similar to Amberlyst 35, with *S*_para_ of 63% for zeolite Y and 54%‐59% for the other catalysts. The *S*_para_ reached with zeolite Y is significantly lower than the 87% reported by Kalechits and Rusak,^[^ ^21^ ^]^ in part because our substrate was a mixture of *cis*‐ and *trans*‐carane instead of pure *cis*‐carane.

For zeolite Y, the effect of the solvent on selectivity and activity was investigated (see SI, Table S1, Supporting Information). The highest *S*_para_ of 63% at 70 °C was observed with non‐polar solvents (alkanes, CHCl_3_) and the solventless isomerization of pure carane. Oxygenated solvents showed a strong inhibiting effect, requiring an increase in reaction temperature up to 100 °C. In acetone and ethylacetate, a *S*_para_ of 61% and 58% was observed, while in ether solvents (diethylether, 1,4‐dioxane), it was lowered to 50%. In methanol, up to 27% of ethers of unknown configuration were formed as byproducts through addition of the solvent to the double bond.

**Figure 3:**
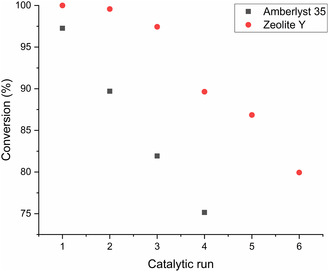
Recycling of Amberlyst 35 and zeolite Y for the isomerization of carane. Conditions: catalyst (acid sites)/substrate: 3.6 mol% (A35), 0.4 mol% (Y).

Recycling experiments were conducted for zeolite Y and Amberlyst 35 (**Figure** **3**). A linear decrease in activity for Amberlyst 35 was observed under solventless conditions, with a reduction of the conversion from initially 97% to 75% in the fourth run. The recycling of zeolite Y was not carried out under solventless conditions because the strongly exothermic reaction causes instant deactivation of the catalyst. In CHCl_3_, full conversion was reached in the first two runs, followed by a decrease to 80% in the next four runs. Thermogravimetric analysis (TGA) of the reused zeolite Y showed a weight loss of 15% after the sixth run, when heated up to 800 °C in an oxidizing atmosphere. This indicates a significant build‐up of organic coke on the catalysts, explaining the deactivation.

The separation of *m*‐ and *p*‐menthenes is difficult due to their similar physical properties. The fractional distillation of the menthene mixture resulted in a good separation of the higher‐boiling isomers **12** and **15** from the other isomers. The fractions with the highest *p*‐3‐menthene content showed an isomer distribution of 59% **4**, 21% **17**, 6% **16**%, and 14% of other isomers and side products.

Overall, the ion exchange resin Amberlyst 35 and zeolite Y showed the best results for the isomerization of carane of all the investigated catalysts, with *p*‐3‐menthene yields of around 50%. Zeolite Y showed a significantly higher activity than the ion exchange resin, but no improvement of selectivity was observed. The significantly higher activity of the zeolite can pose a disadvantage because the use of a solvent is required to control the otherwise strongly exothermic reaction, leading to rapid catalyst deactivation.

### 2.3 Epoxidation of Menthenes

**Scheme 3:**
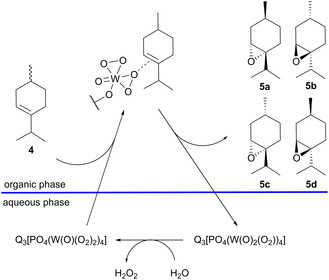
Epoxidation of (+)‐*p*‐3‐menthene with the Ishii‐Venturello‐PTC catalytic system (**Q**: C_25_H_54_N^+^ [Aliquat 336]).

The epoxidation of menthene **4** results in a mixture of 3,4‐epoxymenthane (**5**) isomers. Initial epoxidation experiments of the menthene mixture obtained from carane isomerization (51% **4**, 21% **17**, 11% **16,** 6% **12**, 5% **15**, 6% other) with stoichiometric amounts of *m*‐chloroperbenzoic acid as oxidant gave a yield of 90% for the combined epoxides. The epoxides were stable when stored at room temperature, with no observable change in composition after several months. Next, two different catalytic systems known from the literature have been chosen for investigation. The first system is the Ishii‐Venturello phase transfer (IV‐PTC) system, based on the Venturello anion [PO_4_(W(O)(O_2_)_2_)_4_]^3−^ as the catalytic species and the quaternary ammonium ionic liquid Aliquat 336 as the phase transfer catalyst (see **Scheme** **3**).^[^ ^35^, ^36^, ^37^ ^]^ The second catalytic system uses MTO as catalyst.^[^ ^26^, ^38^ ^]^ Both systems are using the oxidant H_2_O_2_ in a biphasic system.

**Figure 4:**
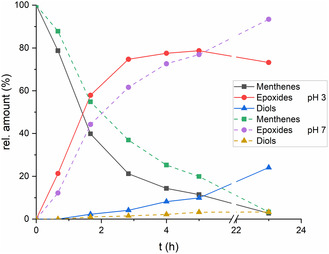
Time‐dependent distribution of products for the epoxidation of a menthene mixture with the IV‐PTC system with variation of the pH of the H_2_O_2_ solution.

The best results were achieved with the IV‐PTC. Here, typically conversions of over 90% and selectivity around 80% for epoxide formation were observed for epoxidations at room temperature with 30 wt% H_2_O_2_ solution. The pH of the added H_2_O_2_ solution has a significant influence on selectivity (**Figure** **4**). When the pure H_2_O_2_ solution with pH 3 was used for the epoxidation, the amounts of epoxides in the reaction mixture stagnated at 75%–80% after 79% conversion because of the pronounced hydrolysis to diols. After 23 h, up to 24% of diols were observed. When an H_2_O_2_ solution with an adjusted pH 7 was used, the hydrolysis rate of the epoxides was lowered, resulting in a higher selectivity of 93% after 23 h. A C/S ratio of 1‐3 mol% proved to give the best results with full conversion after 3 h. At a high C/S ratio of 5 mol%, the conversion after 3 h was only 23%. This is likely caused by the high amount of PTC, resulting in the formation of a stable emulsion and limited diffusion between the organic and aqueous phases.

During the epoxidation of the menthene mixture obtained from carane isomerization, the tetrasubstituted olefins (**12** and **15**) were transformed faster than the trisubstituted olefins (**4**, **16,** and **17**). The *cis*‐ and *trans*‐3,4‐epoxymenthanes (main product) were obtained in a ratio **5a+b**/**5c+d** of 66:34. When the reaction was repeated with a mixture of *p*‐menthenes (70% **4**, 14% **12**, 10% *p*‐1‐menthene, 5% **13**; via hydrogenation‐dehydration of terpineol), the final product mixture contained 85%, **5**%, and 9% *p*‐4,8‐epoxymenthane.

**Figure 5:**
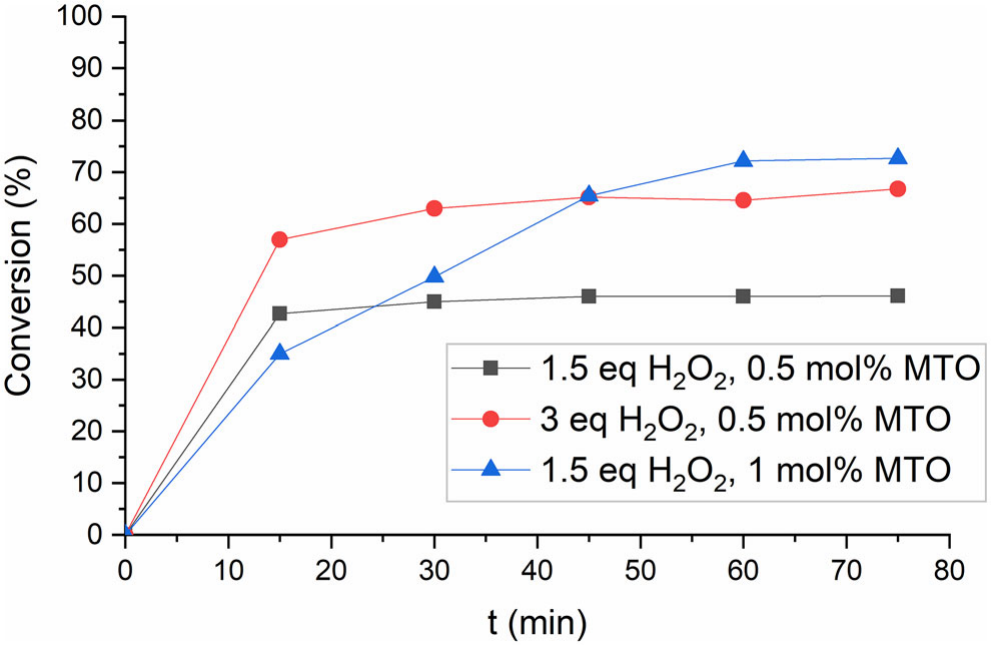
Conversion of menthenes in the epoxidation with MTO catalytic system.

The MTO catalyst system showed high exotherm at pH 7, requiring a cooling of the reaction mixture. The selectivity was >97%, with epoxide hydrolysis being successfully suppressed by the addition of pyridine. At a C/S ratio of 0.5 mol% and 1.5 eq H_2_O_2_, a maximum conversion of 46% was achieved (**Figure** **5**). With a higher C/S ratio of 1 mol%, the maximum conversion increased to 73%, whereas an excess of 3 eq. H_2_O_2_ increased the conversion to 67%. In comparison, Sharpless and Rudolph reported 99% selectivity and full conversion under similar conditions.^[^ ^26^ ^]^ The differences in reaction rates can be related to fluctuating reaction temperature due to inefficient cooling. The high activity in the early stage of the reaction and the following stagnation in conversion indicate a deactivation of the catalyst during the reaction. The decomposition of MTO in general is accelerated by high temperatures, high H_2_O_2_ concentration, and low concentration of suitable basic additives (e.g., pyridine).^[^ ^26^, ^38^ ^]^

Both catalytic systems use the cheap and environmentally friendly oxidant H_2_O_2_. In terms of cost, the IV‐PTC components are cheap and highly available, while being less active than the comparably pricey MTO. With MTO, the hydrolysis is very fast, and organic bases like pyridine have to be used in the system to lower the Lewis acidity of the catalyst. As both systems are homogeneous, the separation and recycling of the catalysts pose a challenge. In this work, the IV‐PTC catalyst was removed from the reaction mixture by filtration over silica and discarded. Cunningham et al. were able to recover and reuse the catalyst from the organic phase by vacuum distillation.^[^ ^37^ ^]^ However, fast loss of activity was reported for this method because of the accumulation of polymeric residues and the leaching of the catalyst into the aqueous phase. The preparation method of the IV‐PTC catalyst requires the use of carcinogenic chlorocarbon solvents. Therefore, alternative systems should be considered for further optimization of the synthesis route. Heterogenized polyoxometalates or systems that change from homogeneous to heterogeneous throughout the reaction should be preferred due to simpler recovery of the catalyst.^[^ ^39^, ^40^ ^]^

### 2.4 Hydrogenation and Isomerization of Epoxides

**Scheme 4:**
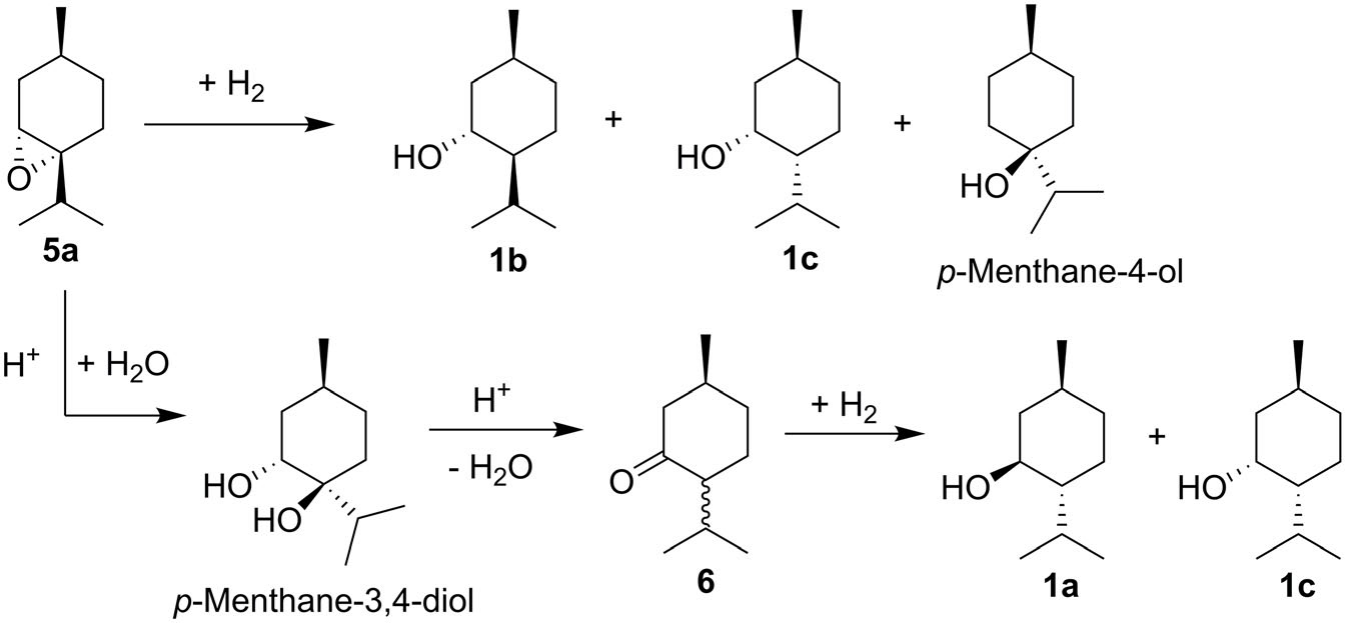
Reaction scheme for the hydrogenation and isomerization of 3,4‐epoxymenthane **5a**.

For the hydrogenation of the epoxides to the corresponding alcohols (**Scheme** **4**), the hydrogenation catalysts Raney‐Ni, Pd/C, Rh/Al_2_O_3,_ and Pt/C were tested with the solvents ethanol and isopropanol. Of all tested catalysts, only Raney‐Ni gave satisfying results. The hydrogenation at 130 °C and 50 bar H_2_ pressure in EtOH resulted in a complete conversion of the epoxide mixture from the previous step to a complex mixture of alcohols, containing 60% of isomenthol (**1b**) and 9% menthol (**1a**). With the other catalysts, no reaction took place at lower temperatures, while at higher temperatures, deoxygenation of the epoxides to menthanes was pronounced. When milder conditions or shorter reaction times were applied, the preferred conversion of the *cis*‐3,4‐epoxymenthanes (**5a, 5b**) was observed in agreement with the literature.^[^ ^28^ ^]^

Because the product of this synthesis route is a racemic mixture of menthol isomers, isomerization to menthol (**1a**) followed by distillation and racemic resolution, analogous to the industrial menthol synthesis route of Symrise,^[^ ^7^ ^]^ is required to obtain the desired (−)‐**1a**. As the process is well known to the authors, it was not further investigated in this work.

As an alternative to direct hydrogenation of the epoxide mixtures, acidic isomerization to the corresponding ketones was attempted. This reaction is known to be catalyzed by a variety of homogeneous Lewis acids, but heterogeneous catalysis is less common.^[^ ^41^ ^]^ Initial experiments with zeolite Y showed mainly decomposition of the epoxides. Surprisingly, the isomerization over the ion exchange resin Amberlyst 35 in *c*‐hexane at 60–80 °C gave a product mixture containing 60% of menthone **6** in good yield, with a ratio of the two stereoisomers menthone/isomenthone of 74:26. However, we found that the reaction is promoted by traces of water in the catalyst, leading to a formation of diols, which then are dehydrated to **6** over the course of the reaction (see Scheme 4). **6** was successfully reduced, via hydrogenation over Raney‐Ni at 100 °C and 80 bar H_2_ to a mixture of 70% **1c**, 27% **1a**, and 3% **1b+d**, or via transfer‐hydrogenation over Raney‐Ni in isopropanol at 150 °C, to a mixture of 50% **1c**, 48% **1a**, and 2% **1b+d**. This approach poses a lower risk of epoxide decomposition and can be a suitable alternative to the direct hydrogenation of epoxides.

## 3 Conclusion

In this article, a new synthesis route for menthol starting from 3‐carene was presented. The synthesis route was designed in accordance with the principles of green chemistry. The starting material is cheap, highly abundant, and sustainable. The route minimizes reaction steps and utilizes environmentally friendly and sustainable reagents like hydrogen and hydrogen peroxide and can, in principle, be solvent‐free. As far as possible, heterogeneous catalysis is applied. This is a significant advantage to the menthol synthesis routes of Takasago and BASF, which heavily rely on homogeneous catalysis. While these homogeneous catalysts are highly active and selective, they are also expensive and difficult to recycle. Our route offers the possibility of utilizing a variety of catalysts. Catalysts like Rh/Al_2_O_3_ can be exchanged for cheaper catalysts like Ru/C or Raney‐Ni with only minor loss of selectivity, which is beneficial for the overall cost efficiency of the process.

In the first reaction step (hydrogenation of 3‐carene to carane), the third (epoxidation of *p*‐3‐menthene to 3,4‐epoxy‐*p*‐menthane) and fourth steps (hydrogenation of the epoxide to menthol), high *S*_chemo_ > 90% was achieved. The main challenge remaining so far is the medium *S*_stereo_ of the isomerization of carane to *p*‐3‐menthene in the second step. Here, a mixture of around 60% *p*‐ and 40% *m*‐menthene isomers is formed, with the *m*‐menthenes being undesired side products. This leads to the maximum theoretical overall yield of the route of around 50% menthol isomers (based on the selectivity of 99% for step 1, 63% for step 2 [with isomer recycling], 93% for step 3%, and 90% for step 4). In comparison, according to Lawrence, the Takasago route accomplishes a yield of around 80% (−)‐menthol, while the Malti‐Chem‐Process (which shares some of the reaction steps with our route) had a yield of ≈25% menthol isomers.^[^ ^1^ ^]^ Therefore, the main goal of future research is to improve the selectivity toward the formation of *p*‐isomers in the second step of the synthesis route. After this step, an efficient separation of the side products has to be established, which so far has proved challenging due to the similar physical properties of the formed isomers. The *m*‐menthenes could be utilized as starting materials for the production of other chemicals like *m*‐menthane, *m*‐cymene, or *m*‐cresol.

As most of the reaction steps are exothermic, the upscaling of the synthesis route will require efficient cooling of the reactors to maintain the selectivity of the transformations. However, the upscaling should not pose significant difficulties, as hydrogenation, acidic isomerization, and epoxidation are already well established in other large industrial‐scale processes. The used catalysts (with exception of the IV‐PTC catalyst) and reagents are commercially available. The catalyst lifetimes will, however, also depend upon the purity of the starting material. Therefore, a prior purification (especially desulfurization) procedure for 3‐carene might have to be developed. Overall, the feasibility of the new route has been demonstrated. Further research is needed to elevate this process to a commercially competitive level.

## Conflict of Interest

The authors declare no conflict of interest.

## Author Contributions

**Dominik Dylong**: conceptualization; investigation; methodology; validation; writing—original draft. **Johannes Panten**: conceptualization; resources; writing—review & editing. **Bernhard Rußbüldt**: conceptualization; resources; writing—review & editing. **Peter J. C. Hausoul**: resources; supervision; writing—review & editing. **Regina Palkovits**: supervision; writing—review & editing. **Matthias Eisenacher**: conceptualization; funding acquisition; project administration; resources; supervision; writing—review & editing.

## Supporting information

Supplementary Material

## Data Availability

The data that support the findings of this study are available in the supplementary material of this article.
