# Supplementary material for: From Turpentine to (−)‐Menthol: A New Approach
Source: ChemSusChem. 2025 Jul 9;18(17):e202500515. doi: 10.1002/cssc.202500515 (PMC12404015; doi:10.1002/cssc.202500515)
Supplement: Supplementary file 1 — Supplementary Material [file CSSC-18-e202500515-s001.pdf]

Supporting information

From Turpentine to (-)-Menthol: a New Approach

Dominik Dylong,[a] Johannes Panten,[b] Bernhard Rußbüldt,[b] Peter J. C. Hausoul,[c] Regina Palkovits,[c,d] and Matthias Eisenacher*[a]

[a] Circular Transformation Lab, TH Köln –University of Applied Sciences, Leverkusen, Germany

[b] Symrise AG, Holzminden, Germany

[c] Institute of Technical and Macromolecular Chemistry, RWTH Aachen University, Aachen, Germany

[d] Institute for a Sustainable Hydrogen Economy, Forschungszentrum Jülich, Jülich, Germany

E-mail: matthias.eisenacher@th-koeln.de

CONTENT

1 Materials and Methods 3

1.1 Reagents and solvents 3

1.2 Batch hydrogenation of 3-carene and epoxymenthanes in Parr-apparatus 3

1.2.1 Recycling of Rh/Al_2O_3 3

1.2.2 Turnover frequencies (TOF), turnover numbers (TON) 3

1.3 Isomerisation of carane with acidic catalysts 4

1.3.1 Recycling experiments: 4

1.4 Preparation of preformed Ishii-Venturello catalyst 4

1.5 Epoxidation of menthenes 4

1.6 Analysis of reaction mixtures and products 5

1.7 Calculation of selectivities 5

2 Characterisation of catalysts 5

2.1 Figure S1: Temperature programmed reduction (TPR-H_2) profiles of supported hydrogenation catalysts Rh/Al_2O_3, Rh/C, Pt/C, Pd/C, Ru/C 7

2.2 Figure S2: Concentration-time profiles of the hydrogenation of 3-carane with the supported hydrogenation catalysts Rh/Al_2O_3 (A), Merck; Rh/C, [Johnson Matthey]; Pt/C (A), [Johnson Matthey], Pd/C, [Acros] Ru/C, [Johnson Matthey]. 8

2.3 Figure S3: Powder XRD diffractograms (Cu-Kα) of zeolites PSH-3 (MWW), ZSM-5 (MFI), Mordenite (MOR), Ferrierite (FER), Y (FAU), Beta (BEA). 9

2.4 Figure S4: Temperature programmed desorption (TPD-NH3) profiles of zeolites PSH-3 (MWW), ZSM-5 (MFI), Mordenite (MOR), Ferrierite (FER), Y (FAU), Beta (BEA). 10

3 Spectroscopic data for products 10

3.1 cis- & trans-carane (**7**) 10

Figure S5: Quantitative 13C NMR spectrum (101 MHz, CDCl3) of a mixture of cis-carane (7) (major isomer) and trans-carane (7) (minor isomer), recorded in inverse gated decoupling mode, with 35 mg/ml of Cr(acac)3. 11

3.2 p-3-Menthene (**4**) 11

3.2.1 Figure S6: Quantitative ^{13}C NMR spectrum (101 MHz, $CDCl_3$) of a mixture of *p*-3-menthene (4) (major isomer), p-1-menthene (minor isomer), p-4(8)-menthene (minor isomer) and p-8-menthene (minor isomer), recorded in inverse gated decoupling mode, with 35 mg/ml of $Cr(acac)_3$. 12

3.3 cis- & trans-3,4-Epoxy-p-menthane (**18**) 12

3.3.1 Figure S7: Quantitative ^{13}C NMR spectrum (101 MHz, $CDCl_3$) of a mixture of *cis*-3,4-epoxy-*p*-menthane (18a) (major isomer) and *trans*-3,4-epoxy-*p*-menthane (minor isomer), with traces of other p-epoxy-menthanes, recorded in inverse gated decoupling mode, with 35 mg/ml of $Cr(acac)_3$. 13

3.4 Isomenthol (**5b**) 13

4 Additional experimental results 14

4.1 Figure S8: Conversion during the isomerisation of carane over selected acidic catalysts. [Reaction conditions: c(carane)= 0.35 mol/l; T= 80 °C; solvent: c-Hex; catalyst (acidic centres)/substrate: 2.7 mol%.] 14

4.2 Figure S9: Influence of the solvent on the activity and selectivity in the hydrogenation of 3-carene with Rh/Al_2O_3. [Reaction conditions: c(Caran)=0.98 mol/l, T=25 °C, $p(H_2)$=15 bar, catalyst: Rh/Al_2O_3 (5%), 0.15 mol%.] 15

4.3 Table S1: Selectivity of the catalytic isomerisation of carane over zeolite Y and Amberlyst 35 under varying reaction conditions. 15

4.4 Figure S10: Turnover frequency of Rh/Al_2O_3 catalyst for the solventless hydrogenation of 3-carene in three consecutive runs. [Reaction conditions: T=25 °C; $p(H_2)$=20–33 bar; catalyst: Rh/Al_2O_3 (5%), 0.1 mol%. Procedure see ESI, section 1.2.1]. 16

1 Materials and Methods

1.1 Reagents and solvents

All reagents and solvents purchased from commercial suppliers were used as received. The declared GC purity of 3-Carene (Acros Organics) was 90%, with tested values of 91–95% for different batches. The purity of all solvents was ≥ 99%.

The hydrogenation catalysts with 5wt% of metal content (Rh/Al_2O_3 (A), Merck; Rh/Al_2O_3 (B), Johnson Matthey; Rh/C, Johnson Matthey; Pt/C (A), Johnson Matthey; Pt/C (B), Acros; Pd/C, Acros; Ru/C, Johnson Matthey) were dried at 80 °C and then reduced in a hydrogen stream for 90 min at 400 °C prior to use. When Raney nickel (Acros Organics, 50% slurry in water) was used for hydrogenations in ethanol, the catalyst suspension was measured in a small beaker, the metal then separated with a magnet at the bottom and the liquid carefully decanted or pipetted and replaced with ethanol (three times).

The zeolite isomerisation catalysts were calcined for 2 h at 550 °C in air prior to use.

1.2 General procedure for batch hydrogenation of 3-carene and epoxymenthanes in Parr-apparatus

Hydrogenation reactions were conducted in 75 ml stainless steel or Hastelloy C high pressure reactors equipped with cross shaped magnetic stirring bars on a Series 5000 Multiple Reactor System from Parr. After addition of the substrate (3-carene or epoxymenthane mixture obtained in previous synthesis step), solvent and catalyst (supported platinum metals or Raney nickel), the reactor was purged three times with nitrogen, pressurised with hydrogen and heated to the reaction temperature, while stirring at 400 rpm. The hydrogen consumption and progress of reaction was monitored via the pressure change in the reactor. The hydrogenation products were analysed via GC(MS) and ^{13}C-NMR. After the reaction, the catalysts were separated via filtration (supported platinum metals) or magnetic separation and decantation (Raney nickel) and the solvent removed *in vacuo*.

1.2.1 Recycling of Rh/Al_2O_3

The hydrogenation was conducted between 33 bar and 20 bar H_2 pressure in the following manner: After addition of the substrate (20 g) and catalyst (0.3 g, 0.1 mol%) the reactor was purged three times with nitrogen, pressurised with hydrogen to 33 bar (± 2 bar) and the reaction started by stirring at 400 rpm. After the pressure dropped to 20 bar (± 1 bar), the reactor was again pressurized to 33 bar (± 2 bar) H_2. This procedure was repeated until the H_2 consumption ceased, indicating full conversion. After the hydrogenation, the catalyst was filtered, rinsed with EtOH, dried at 100 °C, weighed and used in the next reaction cycle.

1.2.2 Turnover frequencies (TOF), turnover numbers (TON)

TOFs and TONs of the hydrogenation reactions of 3-carene (**2**) were calculated from the H_2 pressure data. The overall (mean) TOF and TON of a hydrogenation were calculated as follows:

$$TON = \frac{X_{t_{max}}(\mathbf{2}) * n(\mathbf{2})}{n(metal)}$$

$$TON_{ms} = \frac{TON}{D}$$

$$TOF = \frac{TON}{t_{max}}$$

$$TOF_{ms} = \frac{TOF}{D}$$

with X: conversion, t_{max}: reaction time, n: amount of substance, ms: metal surface atoms, D: dispersion of metal atoms.

The TOF for the recycling experiments for Rh/Al_2O_3 was determined as follows:

$$TOF = \frac{\Delta p(\boldsymbol{H_2}) * V}{\Delta t * R * T * n(metal)}$$

with V: volume of the gaseous phase, R: molar gas constant, T: reaction temperature.

1.3 Isomerisation of carane with acidic catalysts

The isomerisation of carane was conducted mainly in a glass apparatus equipped with a heated oil bath, reflux condenser and magnetic stirrer. Carane (92–94%, impurities: 1,1,4-trimethylcyloheptane < 2%; *p*-menthane < 3%; other < 2%), obtained in the previous synthesis step and different solvents were heated to the reaction temperature and the reaction was started by addition of the catalyst. During the reaction, samples were taken periodically and analysed via GC to monitor the progress of the isomerisation.

Reactions above the boiling point of the used solvents were conducted in 75 ml Hastelloy C high pressure reactors equipped with cross shaped magnetic stirring bars on a Series 5000 Multiple Reactor System from Parr under nitrogen atmosphere.

1.3.1 Recycling experiments

After the reaction, the catalyst was filtered, rinsed with solvent, dried at 100 °C, weighed and used in the next reaction.

1.4 Preparation of preformed Ishii-Venturello catalyst

The catalyst was prepared based on the method described by Cunningham et al. (W. B. Cunningham, J. D. Tibbetts, M. Hutchby, K. A. Maltby, M. G. Davidson, U. Hintermair, P. Plucinski and S. D. Bull, *Green Chem.*, 2020, **22**, 513–524.) Tungstic acid (18 g, 72 mmol) and 60 wt% aqueous H_2O_2 (20 mL) solution, diluted with 30 ml distilled H_2O, were stirred for 90 min at 60 °C. The mixture was cooled to room temperature and filtered to remove nonsoluble aggregates. A solution of 85% phosphoric acid (H_3PO_4) (2.23 mL) in distilled H_2O (2.23 mL) was added, followed by addition of 180 mL of distilled H_2O. The reaction mixture was stirred for 30 min at rt, followed by dropwise addition of a solution of Aliquat 336 (16.4 ml) in CH_2Cl_2 (290 mL) over a period of 10 min. The resulting mixture was then stirred vigorously at room temperature for 1 h. The clear organic phase was separated, washed with distilled H_2O, dried with $MgSO_4$ and concentrated under vacuum to give the catalyst as a viscous, transparent, lightly yellow syrup (21.2 g, 75%).

1.5 Epoxidation of menthenes with preformed Ishii-Venturello-PTC system

The epoxidation of the menthene mixtures were conducted in a glass apparatus equipped with a heated oil bath and a reflux condenser. First, the catalyst was dissolved in the substrate (menthene mixture, typically 93%) under stirring, followed by the addition of aqueous hydrogen peroxide solution (30-60 wt%). Prior to addition, the pH of the hydrogen peroxide solution was adjusted to the desired value with aqueous sodium hydroxide. The stirring rate was adjusted, until the organic and the aqueous phase formed an emulsion. The progress of the reaction was monitored periodically by GC. After the reaction, the organic phase was separated and filtrated over silica to remove the catalyst. The crude product was used in the next step as obtained or purified by column chromatography, resulting in a mixture of epoxides with a GC purity of around 90%.

1.6 Epoxidation of menthenes over MTO

The catalyst methyltrioxorhenium(VII) (18 mg, 72 µmol) was dissolved in 1 ml of CH_2Cl_2, followed by addition of pyridine (145 mg, 1.8 mmol) and the menthene mixture (2 g, 14 mmol). Then 30wt% H_2O_2 solution (2.5 ml, 1.5 eq., pH 7) was added and the reaction mixture stirred for 75 min, while beeing cooled with an ice bath. The progress of the reaction was monitored by GC. Consecutive reactions were performed either with the doubled amount of catalyst (36 mg, 144 µmol) or with 3 eq. of 30wt% H_2O_2 solution (5 ml).

1.7 Analysis of reaction mixtures and products

The reaction mixtures and products were analysed via GC/MS on a Shimadzu 2010 gas chromatograph, equipped with an FID detector and a mass spectrometer, on the column Optima 624 (6 % Cyanopropylphenyl - 94 % Dimethylpolysiloxan, 30 m, 0.32 mm ID, 1.80 µm d_f) from Macherey-Nagel.

The ^{1}H- and ^{13}C-NMR spectra were recorded on a Bruker Ascend 400 spectrometer in deuterated chloroform. For quantitative ^{13}C-NMR, 35 mg/ml chromium(III) acetylacetonate was added to the sample solution as a relaxation agent and the spectra were recorded in inverse gated decoupling mode.

1.8 Calculation of selectivities

The chemoselectivity (S_{chemo}) and stereoselectivity (S_{stereo}) for hydrogenation of 3-carene (**2**) were defined as follows:

$$S_{chemo} = \frac{S(cis\mathbf{7}) + S(trans\mathbf{7})}{S(all\ products)}$$

$$S_{stereo} = \frac{S(cis\mathbf{7})}{S(cis\mathbf{7}) + S(trans\mathbf{7})}$$

The S_{chemo} and S_{stereo} for isomerisation of carane (**7**) were defined as follows:

$$S_{chemo} = \frac{S(\mathbf{4}) + S(\mathbf{12\text{-}17})}{S(all\ products)}$$

$$S_{para} = \frac{S(\mathbf{4}) + S(\mathbf{12}) + S(\mathbf{13})}{S(\mathbf{4}) + S(\mathbf{12\text{-}17})}$$

The S_{chemo} for epoxidation of the menthenes was defined as follows:

$$S_{chemo} = \frac{S(\boldsymbol{Epoxides})}{S(all\ products)}$$

2 Characterisation of catalysts

The N_2 adsorption experiments were conducted on an ASAP 2060 (Micromeritics) at -196 °C. The specific surface areas of the heterogeneous catalysts were determined using the Brunauer-Emmett-Teller (BET) method in the range of p/p^0= 0.05-0.3. The micropore surface area was determined via the t-plot method. The metal surface area of the supported metal catalysts was measured via CO pulse chemisorption on a MicroActive AutoChem HP 2950 (Micromeritics). The catalyst samples were first reduced for 30 min at 200 °C in 5% H_2/Ar stream, followed by 30 min of desorption at 200 °C in He stream and followed by the CO pulse titration at 35 °C with 50% CO/He. The dispersion of the metal (D) and mean diameter of the metal nanoparticles (d_{NP}) was calculated as follows:

$$D = \frac{\Delta CO}{n(metal)}$$

$$d_{NP} = \frac{6 * m(metal) * \rho(metal)}{n(metal) * A(metal) * D}$$

The temperature programmed reduction (TPR) of the supported metal catalysts was conducted on a MicroActive AutoChem HP 2950 (Micromeritics). The catalyst samples were pretreated for 30 min at 200 °C in a He stream, followed by reduction in 5% H_2/Ar, applying a temperature ramp of 10 °C/min in the range of 35 °C to 600 °C. The temperature programmed desorption of NH_3 of the zeolites was conducted on a MicroActive AutoChem HP 2950 (Micromeritics). The catalyst samples were first pretreated for 30 min at 500 °C in a He stream, followed by NH_3 adsorption at 50 °C. The desorption was conducted between 50 °C and 500 °C at a heating rate of 10 °C/min. For the calculation of acid sites, only NH_3 desorption in the region between 150 °C and 500 °C was taken into account, as below 150 °C mostly physisorption occurs. The structures of the zeolites were verified via X-ray diffraction (powder XRD) on a D2 Phaser (Bruker) using Cu-Kα radiation (λ=0.154184 nm). The diffractograms were collected in the 5°-50° 2Θ range and at 0.012° intervals.

2.1 Figure S1: Temperature programmed reduction (TPR-H_2) profiles of supported hydrogenation catalysts Rh/Al_2O_3, Rh/C, Pt/C, Pd/C, Ru/C

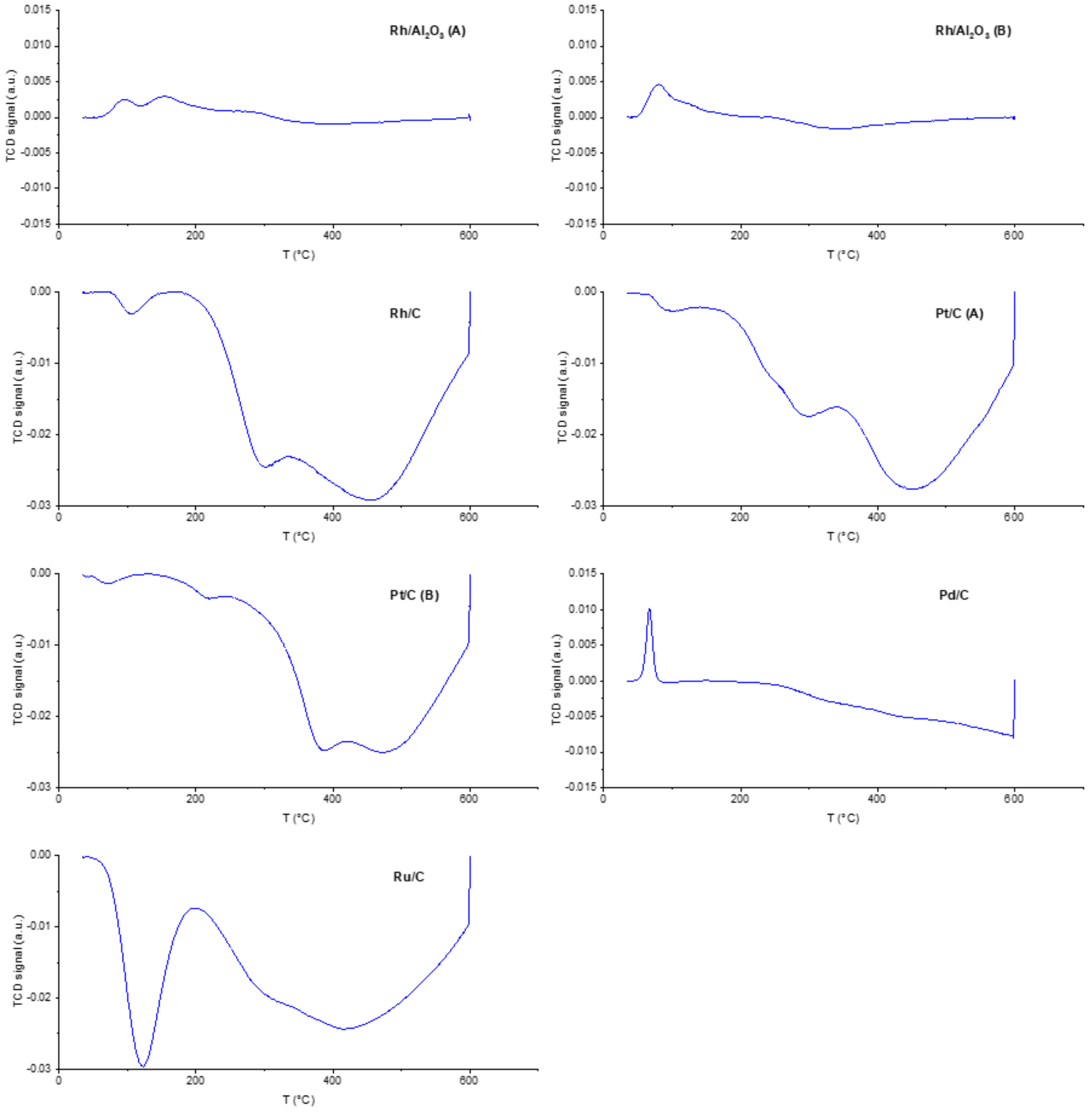

Figure S1: Temperature programmed reduction (TPR-H_2) profiles of supported hydrogenation catalysts Rh/Al_2O_3 (A), Merck; Rh/Al_2O_3 (B), [Johnson Matthey]; Rh/C, [Johnson Matthey]; Pt/C (A), [Johnson Matthey], Pt/C (B), [Acros], Pd/C, [Acros] Ru/C, [Johnson Matthey].

2.2 Figure S2: Concentration-time profiles of the hydrogenation of 3-carane with the supported hydrogenation catalysts Rh/Al_2O_3 (A), Merck; Rh/C, [Johnson Matthey]; Pt/C (A), [Johnson Matthey], Pd/C, [Acros] Ru/C, [Johnson Matthey].

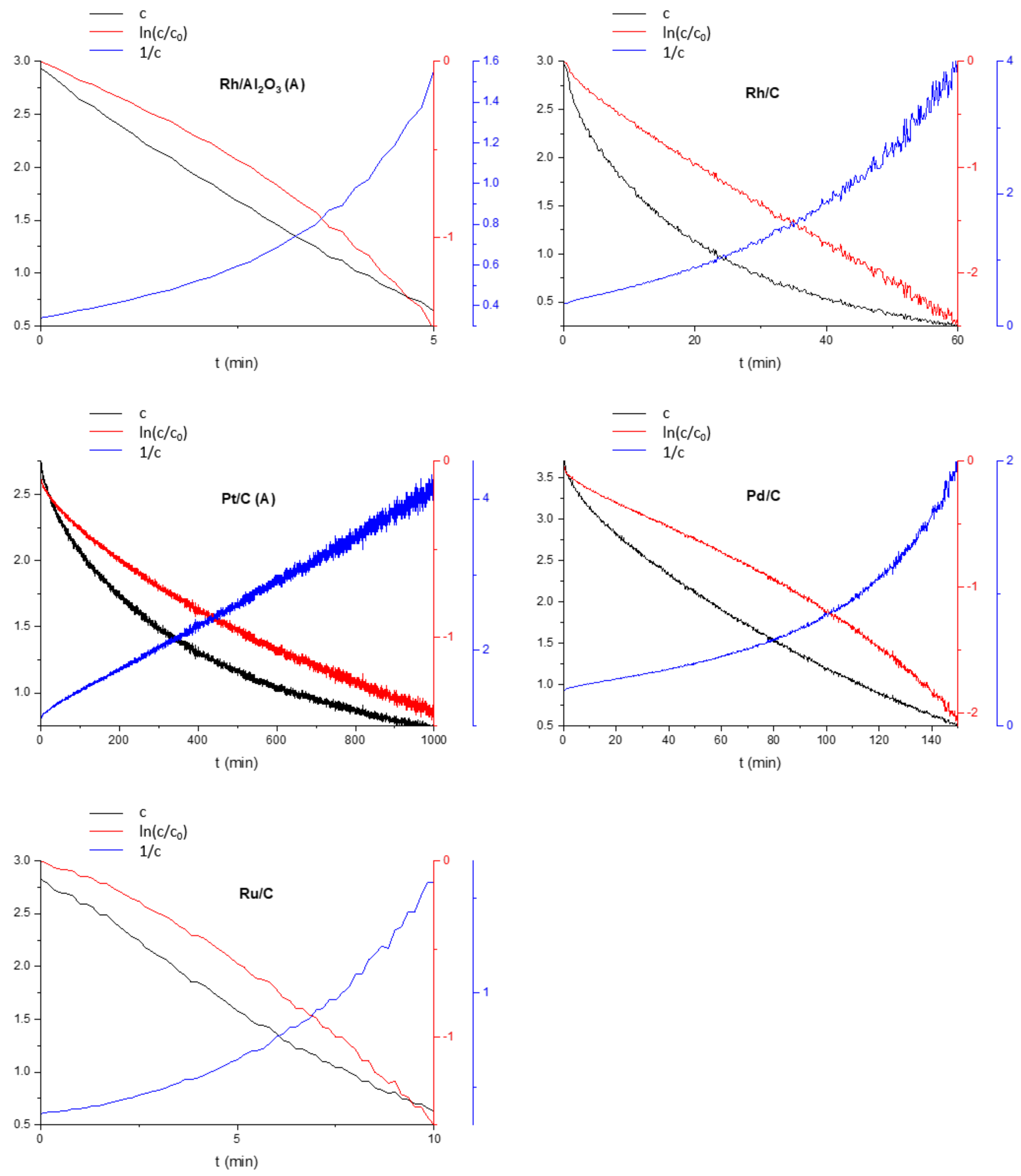

Figure S2: Concentration-time profiles of the hydrogenation of 3-carane with the supported hydrogenation catalysts Rh/Al_2O_3 (A), Merck; Rh/C, [Johnson Matthey]; Pt/C (A), [Johnson Matthey], Pd/C, [Acros] Ru/C, [Johnson Matthey].

2.3 Figure S3: Powder XRD diffractograms (Cu-Kα) of zeolites PSH-3 (MWW), ZSM-5 (MFI), Mordenite (MOR), Ferrierite (FER), Y (FAU), Beta (BEA).

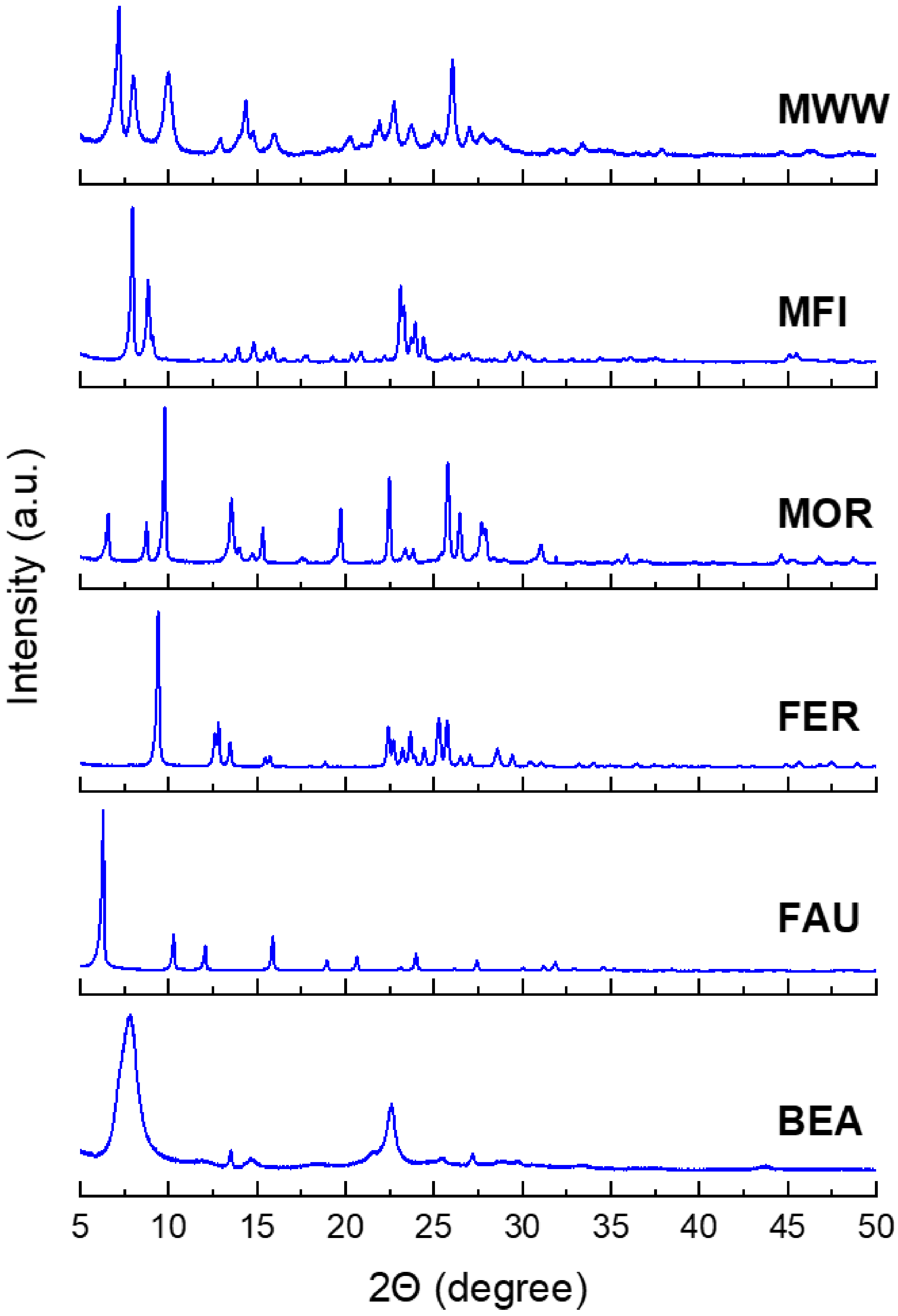

Figure S3: Powder XRD diffractograms (Cu-Kα) of zeolites PSH-3 (MWW), ZSM-5 (MFI), Mordenite (MOR), Ferrierite (FER), Y (FAU), Beta (BEA).

2.4 Figure S4: Temperature programmed desorption (TPD-NH3) profiles of zeolites PSH-3 (MWW), ZSM-5 (MFI), Mordenite (MOR), Ferrierite (FER), Y (FAU), Beta (BEA).

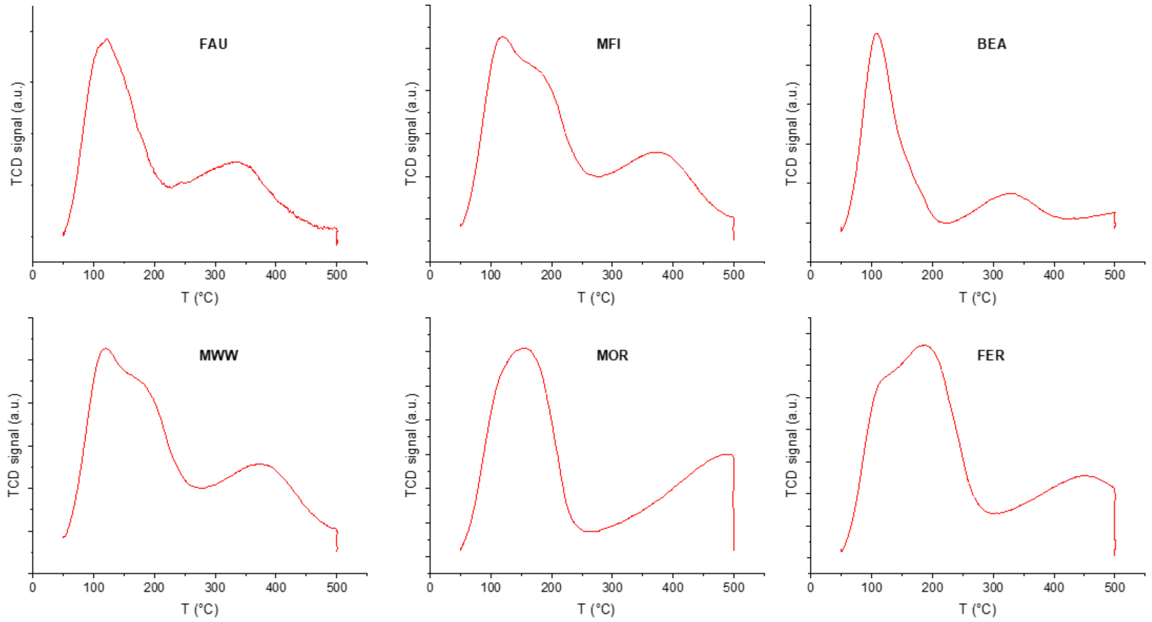

Figure S4: Temperature programmed desorption (TPD-NH_3) profiles of zeolites PSH-3 (MWW), ZSM-5 (MFI), Mordenite (MOR), Ferrierite (FER), Y (FAU), Beta (BEA).

3 Spectroscopic data for products

Note: ^{13}C NMR chemical shifts are referenced to the $CDCl_3$ solvent signal (77.16 ppm). Quantitative ^{13}C NMR data (denoted as q-^{13}C NMR) were measured in *inverse gated decoupling* mode with addition of 35 mg/ml of $Cr(acac)_3$ to the deuterated solvent. Under this conditions, a signal shift of around 0.5 ppm between standard and quantitative experiments was observed. The structural formulae shown represent the diastereomerism, but not the enantiomerism of the characterised compounds.

If products could not be separated via distillation or column chromatography, the ^{13}C NMR signals of the mixtures were assigned based on their peak area (from quantitative ^{13}C NMR) according to the proportion of isomers determined via GC and their multiplicity (from ^{13}C ATP NMR).

3.1 cis- & trans-carane (**7**)

3,7,7-Trimethylbicyclo[4.1.0]heptane

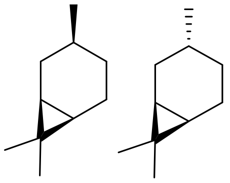

cis-**7** trans-**7**

cis-**7**: ^{13}C NMR (101 MHz, $CDCl_3$) δ [ppm]: 31.37, 29.56, 28.88, 28.61, 22.37, 21.13, 20.01, 18.26, 17.53, 15.60.

trans-**7:** ^{13}C NMR (101 MHz, $CDCl_3$) δ [ppm]: 31.19, 29.64, 28.55, 28.18, 23.05, 19.85, 19.61, 19.21, 17.49, 15.64.

Figure S5: Quantitative 13C NMR spectrum (101 MHz, CDCl3) of a mixture of cis-carane (7) (major isomer) and trans-carane (7) (minor isomer), recorded in inverse gated decoupling mode, with 35 mg/ml of Cr(acac)3.

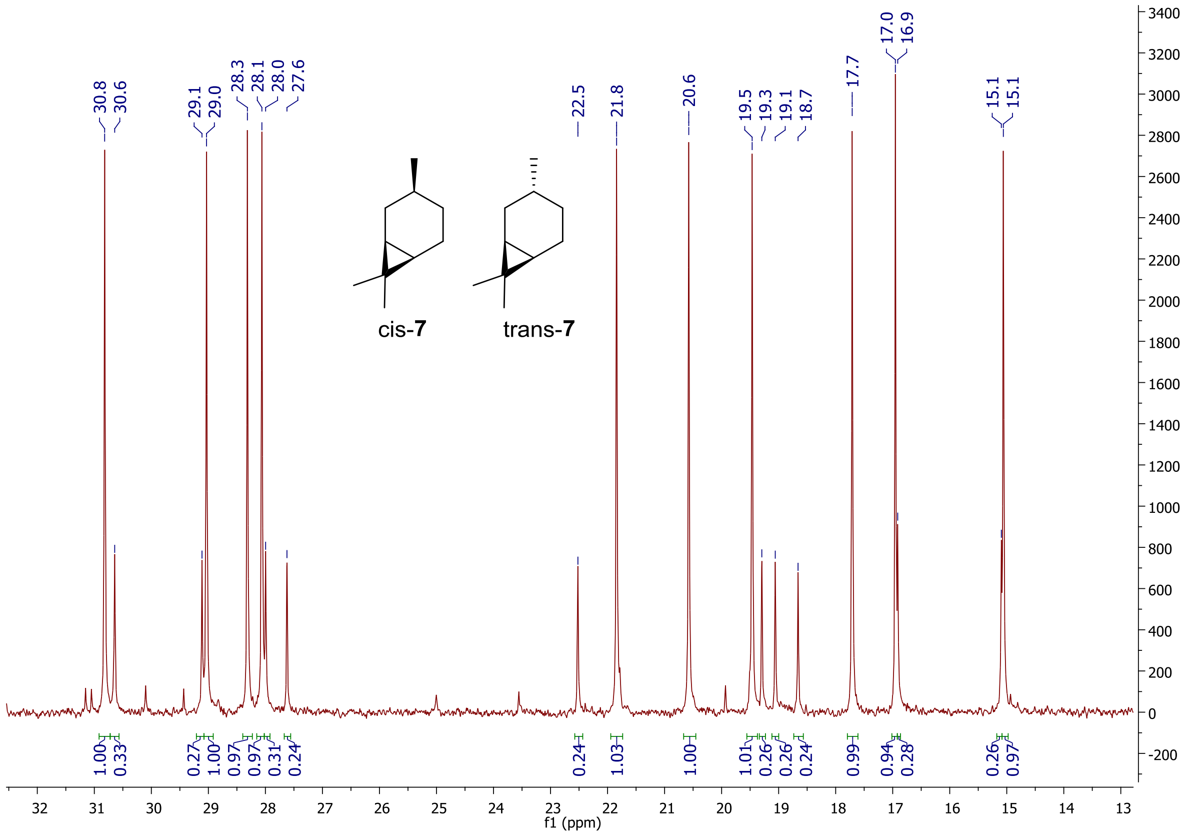

Figure S5: Quantitative ^{13}C NMR spectrum (101 MHz, CDCl3) of a mixture of cis-carane (major isomer) and trans-carane (minor isomer), recorded in inverse gated decoupling mode, with 35 mg/ml of Cr(acac)3.

3.2 p-3-Menthene (**4**)

1-Isopropyl-4-methylcyclohex-1-ene

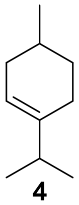

4

4: q-^{13}C NMR (101 MHz, $CDCl_3$) δ [ppm]: 142.66, 117.42, 34.55, 33.48, 31.00, 28.29, 25.57, 21.43, 21.18, 20.82.

3.2.1 Figure S6: Quantitative ^{13}C NMR spectrum (101 MHz, $CDCl_3$) of a mixture of *p*-3-menthene (4) (major isomer), p-1-menthene (minor isomer), p-4(8)-menthene (minor isomer) and p-8-menthene (minor isomer), recorded in inverse gated decoupling mode, with 35 mg/ml of $Cr(acac)_3$.

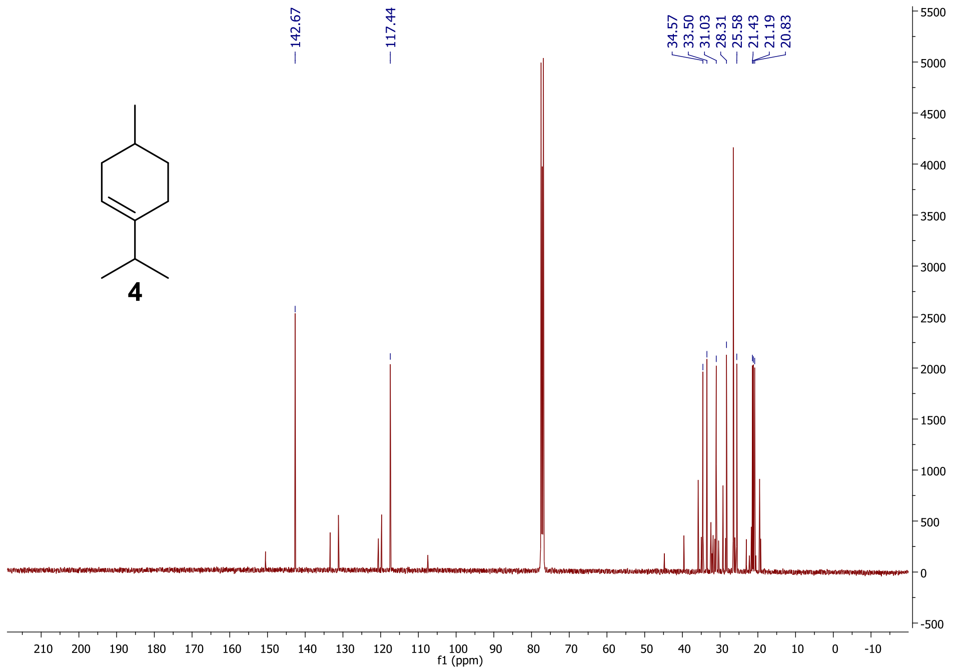

Figure S6: Quantitative ^{13}C NMR spectrum (101 MHz, CDCl3) of a mixture of p-3-menthene (4) (major isomer), p-1-menthene (minor isomer), p-4(8)-menthene (minor isomer) and p-8-menthene (minor isomer), recorded in inverse gated decoupling mode, with 35 mg/ml of Cr(acac)3.

3.3 cis- & trans-3,4-Epoxy-p-menthane (5)

4-Methyl-1-(1-methylethyl)-7-oxabicyclo[4.1.0]heptane

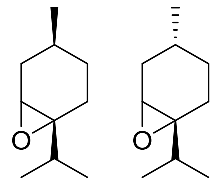

5a **5c**

5a: q-^{13}C NMR (101 MHz, $CDCl_3$) δ [ppm]: 62.81, 58.78, 35.10, 33.79, 29.52, 24.12, 22.84, 20.99, 17.67, 16.49.

5c: q-^{13}C NMR (101 MHz, $CDCl_3$) δ [ppm]: 63.12, 57.75, 34.06, 32.73, 27.82, 26.93, 23.38, 21.45, 17.92, 17.20.

3.3.1 Figure S7: Quantitative ^{13}C NMR spectrum (101 MHz, $CDCl_3$) of a mixture of *cis*-3,4-epoxy-*p*-menthane (5a) (major isomer) and *trans*-3,4-epoxy-*p*-menthane (5c) (minor isomer), with traces of other p-epoxy-menthanes, recorded in inverse gated decoupling mode, with 35 mg/ml of $Cr(acac)_3$.

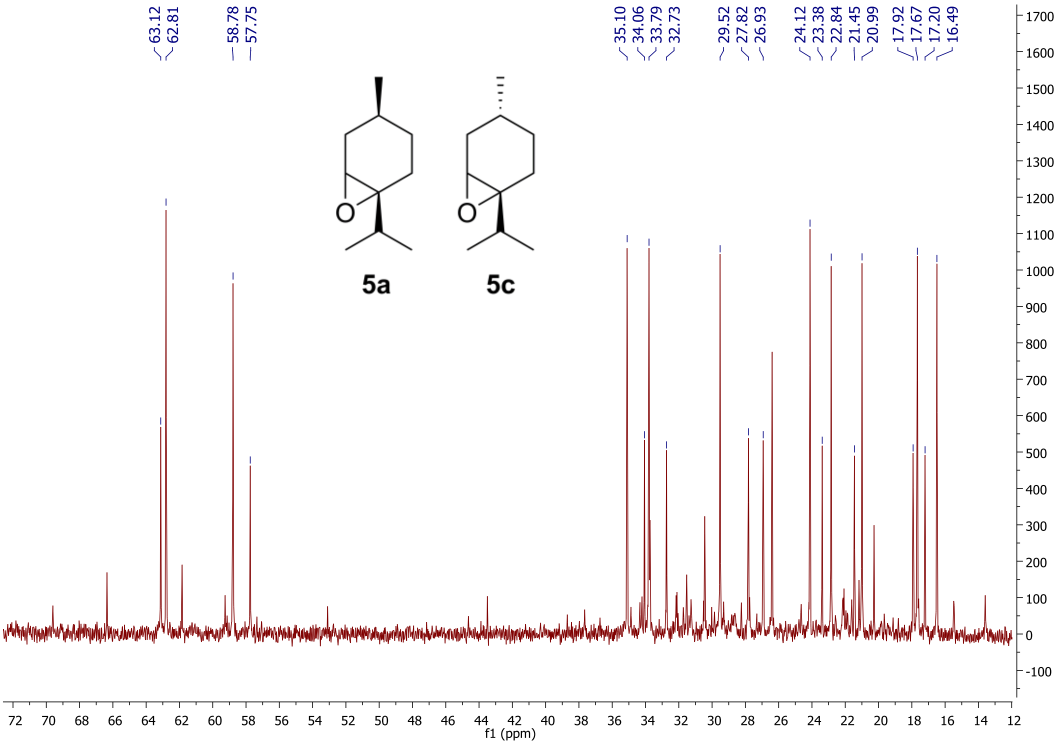

Figure S7: Quantitative ^{13}C NMR spectrum (101 MHz, CDCl3) of a mixture of cis-3,4-epoxy-p-menthane (5a) (major isomer) and trans-3,4-epoxy-p-menthane (5c) (minor isomer), with traces of other p-epoxy-menthanes, recorded in inverse gated decoupling mode, with 35 mg/ml of Cr(acac)$_3$.

3.4 Isomenthol (**1b**)

5-Methyl-2-(1-methylethyl)cyclohexanol

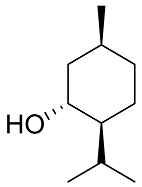

1b

1b: q-^{13}C NMR (101 MHz, $CDCl_3$) δ [ppm]: 67.33, 49.05, 39.55, 30.00, 27.04, 25.50, 20.57, 19.46, 19.01, 17.60.

4 Additional experimental results

4.1 Figure S8: Conversion during the isomerisation of carane over selected acidic catalysts. [Reaction conditions: c(carane)= 0.35 mol/l; T= 80 °C; solvent: c-Hex; catalyst (acidic centres)/substrate: 2.7 mol%.]

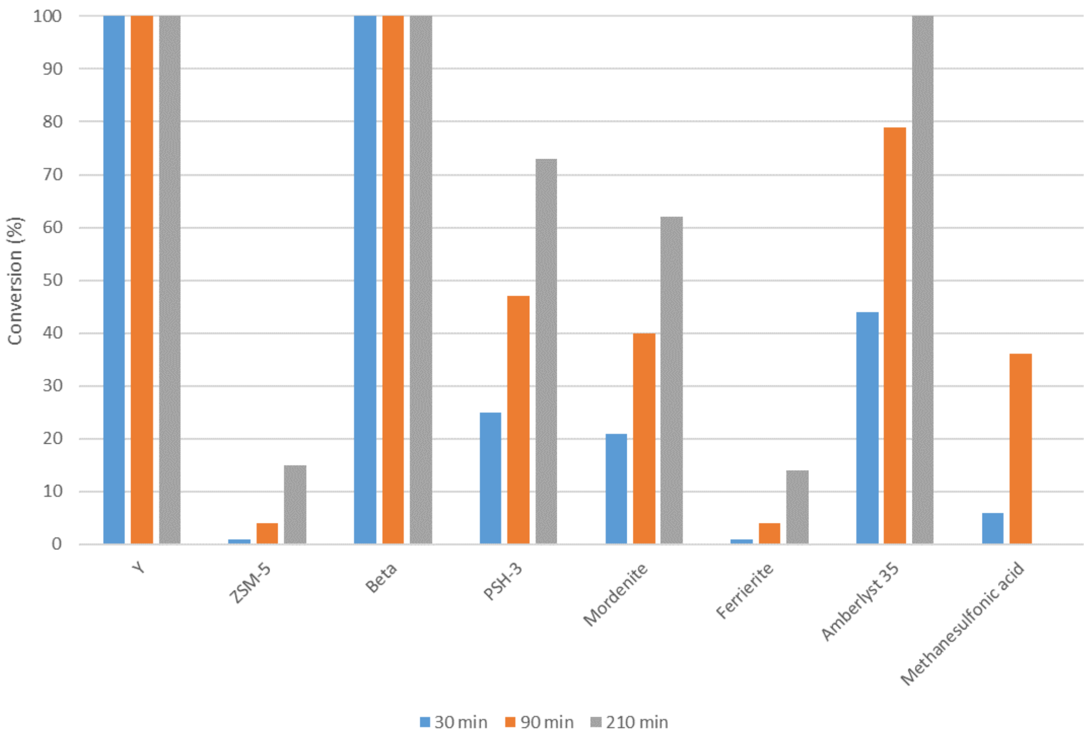

Figure S8: Conversion during the isomerisation of carane over selected acidic catalysts.
Reaction conditions: c(carane)= 0.35 mol/l; T= 80 °C; solvent: c-Hex; catalyst (acidic centres)/substrate: 2.7 mol%.

4.2 Figure S9: Influence of the solvent on the activity and selectivity in the hydrogenation of 3-carene with Rh/Al_2O_3. [Reaction conditions: c(Caran)=0.98 mol/l, T=25 °C, $p(H_2)$=15 bar, catalyst: Rh/Al_2O_3 (5%), 0.15 mol%.]

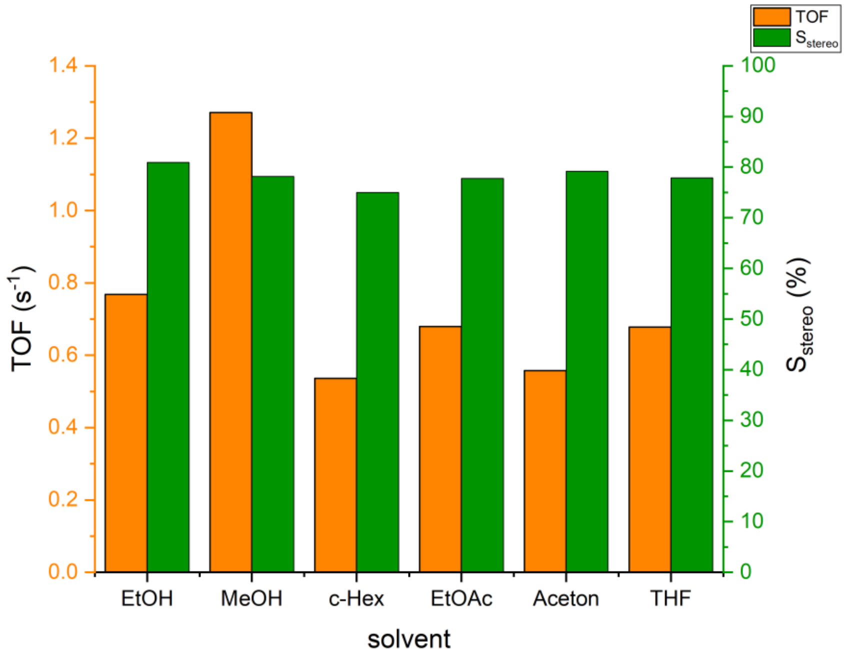

Figure S9: Influence of the solvent on the activity and selectivity in the hydrogenation of 3-carene with Rh/Al_2O_3. Reaction conditions: c(Caran)=0.98 mol/l, T=25 °C, $p(H_2)$=15 bar, catalyst: Rh/Al_2O_3 (5%), 0.15 mol%.

4.3 Table S1: Selectivity of the catalytic isomerisation of carane over zeolite Y and Amberlyst 35 under varying reaction conditions.

Table S1: Selectivity of the catalytic isomerisation of carane over zeolite Y and Amberlyst 35 under varying reaction conditions.

catalyst	solvent	T (°C)	S_{para} (%)	$S_{p\text{-}3\text{-}M}$ (%)
Zeolite Y	n-Hexane	70	63	52
Zeolite Y	n-Heptane	70	63	52
Zeolite Y	c-Hexane	70	63	52
Amberlyst 35	c-Hexane	70	63	53
Zeolite Y	no solvent	70	63	49
Zeolite Y	Chloroform	70	61	50
Zeolite Y	Diethyl ether	100	51	42
Zeolite Y	1,4-Dioxane	100	50	41
Amberlyst 35	1,4-Dioxane	100	61	52
Zeolite Y	Acetone	100	61	50
Zeolite Y	Ethyl acetate	100	58	46

4.4 Figure S10: Turnover frequency of Rh/Al_2O_3 catalyst for the solventless hydrogenation of 3-carene in three consecutive runs. [Reaction conditions: T=25 °C; $p(H_2)$=20–33 bar; catalyst: Rh/Al_2O_3 (5%), 0.1 mol%. Procedure see ESI, section 1.2.1].

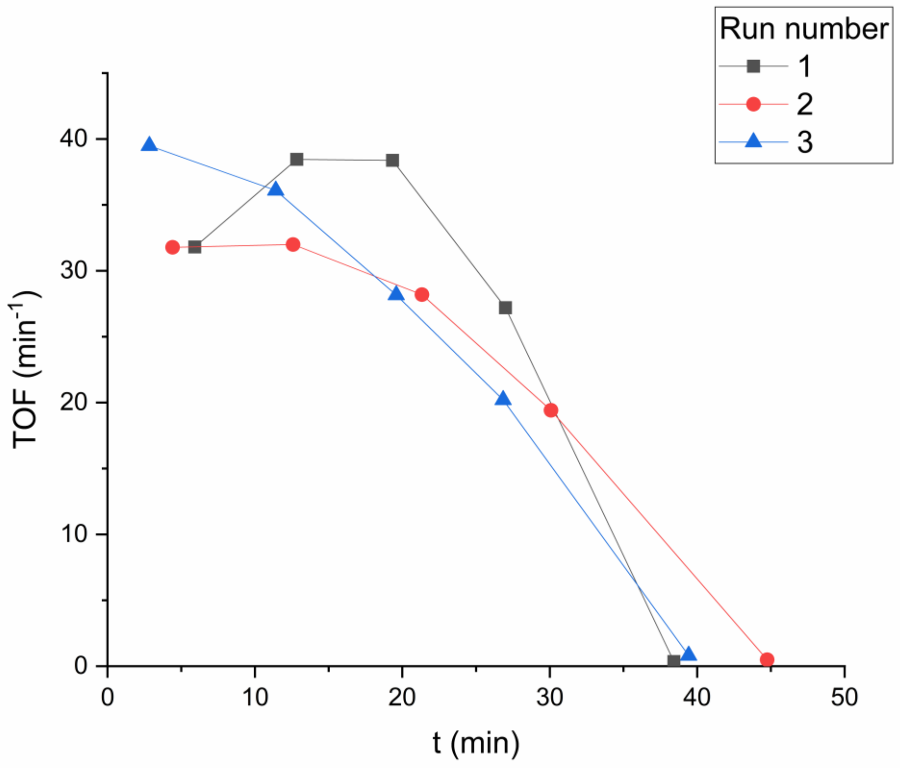

Figure S10: Turnover frequency of Rh/Al_2O_3 catalyst for the solventless hydrogenation of 3-carene in three consecutive runs. [Reaction conditions: T=25 °C; $p(H_2)$=20–33 bar; catalyst: Rh/Al_2O_3 (5%), 0.1 mol%. Procedure see ESI, section 1.2.1].

4.5 Table S2. Comparison of literature results of the isomerisation of carane and *p*-menthenes over various catalysts.

Table S2. Comparison of literature results of the isomerisation of carane and *p*-menthenes over various catalysts.

Catalyst [Ref.]	Substrate	T (°C)	Product distribution of menthenes [M] (%)							
			p-8-M (**13**)	*p*-4(8)-M (**12**)	*p*-3-M (**4**)	*m*-8-M (**14**)	*m*-3(8)-M (**15**)	*m*-2-M (**16**)	*m*-3-M (**17**)	*p*-1-M
Zeolite Y (**this work**)	*cis-/trans-*Carane (84:16)	70	2.0	6.7	54.9	0.2	5.5	10.0	20.6	0
Zeolite Y [1]	*cis*-Carane	100	3.6	8.2	78.8	0	5.2	1.7	2.5	-
Zeolite Y [1]	*trans*-Carane	100	1.2	3.4	30.3	3.3	17.0	17.3	27.6	-
Tseokar-2 [1]	*cis*-Carane	80	4.1	6.3	76.8	0	5.5	2.0	5.3	-
p-Toluenesulfonic acid [2]	*cis*-Carane	80	7.1	6.8	61.0	1.5	5.3	7.4	10.9	-
Co/Al_2O_3 [3]	*p*-Menthenes	250	-	10.8	56.3	-	-	-	-	33.0

References:

[1] G. V. Kalechits, M. F. Rusak, "Transformations of cis- and trans-caranes in the presence of zeolite catalysts" *Zhurnal Obshchei Khimii* **1986**, *56*, 2132.

[2] I. I. Bardyshev, G. V. Deshits, "Acid isomerization of cis- and trans-caranes [abstract]" *Vestsi Akademii Navuk BSSR, Seryya Khimichnykh Navuk* **1975**, 89.

[3] Z. A. Filippenko, G. N. Roganov, V. V. Bazyl'chik, "Equilibrium isomerization of p-menthenes" *Khimiya Prirodnykh Soedinenii* **1988**, 878.
